# Fine-Scale Vertical Stratification and Guild Composition of Saproxylic Beetles in Lowland and Montane Forests: Similar Patterns despite Low Faunal Overlap

**DOI:** 10.1371/journal.pone.0149506

**Published:** 2016-03-15

**Authors:** Matthias Weiss, Jiří Procházka, Jiří Schlaghamerský, Lukas Cizek

**Affiliations:** 1 Biology Centre CAS, Institute of Entomology, České Budějovice, Czech Republic; 2 University of South Bohemia, Faculty of Science, Branišovská 31, 370 05, České Budějovice, Czech Republic; 3 Masaryk University, Faculty of Science, Department of Botany and Zoology, Kotlářská 2, 611 37, Brno, Czech Republic; Università degli Studi di Napoli Federico II, ITALY

## Abstract

**Objective:**

The finer scale patterns of arthropod vertical stratification in forests are rarely studied and poorly understood. Further, there are no studies investigating whether and how altitude affects arthropod vertical stratification in temperate forests. We therefore investigated the fine-scale vertical stratification of diversity and guild structure of saproxylic beetles in temperate lowland and montane forests and compared the resulting patterns between the two habitats.

**Methods:**

The beetles were sampled with flight intercept traps arranged into vertical transects (sampling heights 0.4, 1.2, 7, 14, and 21 m). A triplet of such transects was installed in each of the five sites in the lowland and in the mountains; 75 traps were used in each forest type.

**Results:**

381 species were collected in the lowlands and 236 species in the mountains. Only 105 species (21%) were found at both habitats; in the montane forest as well as in the lowlands, the species richness peaked at 1.2 m, and the change in assemblage composition was most rapid near the ground. The assemblages clearly differed between the understorey (0.4 m, 1.2 m) and the canopy (7 m, 14 m, 21 m) and between the two sampling heights within the understorey, but less within the canopy. The stratification was better pronounced in the lowland, where canopy assemblages were richer than those near the forest floor (0.4 m). In the mountains the samples from 14 and 21 m were more species poor than those from the lower heights. The guild structure was similar in both habitats.

**Conclusions:**

The main patterns of vertical stratification and guild composition were strikingly similar between the montane and the lowland forest despite the low overlap of their faunas. The assemblages of saproxylic beetles were most stratified near ground. The comparisons of species richness between canopy and understorey may thus give contrasting results depending on the exact sampling height in the understorey.

## Introduction

Understanding the patterns of distribution of organisms on various scales is one of the fundamental questions of current ecology. It is also essential for biodiversity conservation, forestry and agriculture. Altitude is among the most prominent factors influencing the distribution of organisms due to its effect on abiotic factors such as climate [1] and soil conditions [2,3]. Distinct turnover in community composition thus often occurs along altitudinal gradients [4,5,6]. In arthropod communities, species richness mostly decreases with elevation [7,8]; it may, however, also increase or exhibit a mid-elevation peak [9,10].

Forests are three-dimensional habitats where organisms are also distributed along the vertical gradient between forest floor and tree tops [11]. Depending on the type of forest and taxa studied, the vertical gradient in stratification of arthropod assemblages might be imperceptible, or it may result in a clear stratification between sampling heights [12,13,14,15,16]. The stratification patterns also change with latitude as stratification is more pronounced in tropical forests than in temperate ones, probably due to the higher complexity of the vertical structure of tropical forests [11]. However, the effect of other geographical factors, such as elevation above sea level on the patterns of arthropod vertical stratification, has never been studied.

Insects associated with the wood of dead or live trees (i.e. saproxylic insects), especially beetles, constitute a substantial portion of forest biodiversity. Owing to their ability to weaken or kill trees and start the decomposition process, many beetles are considered essential components of forest dynamics in the natural forest and serious pests in production forests [17,18]. The recent decrease in the amount of dead wood and old trees in forests has caused serious decline of numerous species [19,20]. Saproxylic beetles are thus intensively studied due to their status as pests or target species of nature conservation [21,22,23]. They also serve as model organisms for identifying sustainable forest management practices [19,24,25].

Despite numerous studies on the ecology of saproxylic beetles, very little is known about their response to altitude. One study [26] reported a decrease in species richness of bark beetles and their associates with altitude, while another [27] reported shifts in community composition of bark beetles between lowland and montane forests. Patterns of diversity and guild structure between lowland and montane forests thus remain largely unknown for the group.

Although the distribution of saproxylic beetles along the vertical gradient in temperate forests has received much attention, a number of issues remain to be solved. Saproxylic beetles are generally considered more abundant and diverse in the understorey of temperate forests, they show a clear vertical stratification and the canopy fauna is not a simple subset of the understorey fauna [28,29,14,30,31,32]. Although several authors [33,11] emphasised the importance of sampling insects along genuine vertical transects, most studies on the vertical distribution of saproxylic beetles have compared two sampling heights only (*cf*. [31]). Limited numbers, or a lack of replicates, limit the information value of studies investigating stratification on a finer scale [33,13,14]. One particular study [16] used an experimental design that was practically identical to ours (see Method section) but studied the entire arthropod community. As such, we still lack authoritative information on the distribution patterns of saproxylic beetles along genuine vertical gradients in temperate forests.

To address the above issues, we sampled saproxylic beetles along a fine-scale vertical gradient in temperate montane and lowland forests. We investigated patterns of assemblage composition, diversity, and feeding guild distribution along a vertical gradient in lowland and montane forests and compare their patterns between the two habitats. Specifically, we aimed to answer the following questions: (i) Is there a difference in the overall diversity and/or guild structure of the beetle assemblages between montane and lowland forests? (ii) Do the patterns of vertical stratification differ between montane and lowland forests? (iii) Is the change in composition of beetle assemblages between understorey and canopy gradual or rather sudden? (iv) How are the feeding guilds distributed among the sampling heights and are the distribution patterns identical in both forest types?

## Methods

### Study sites

**Fig 1:**
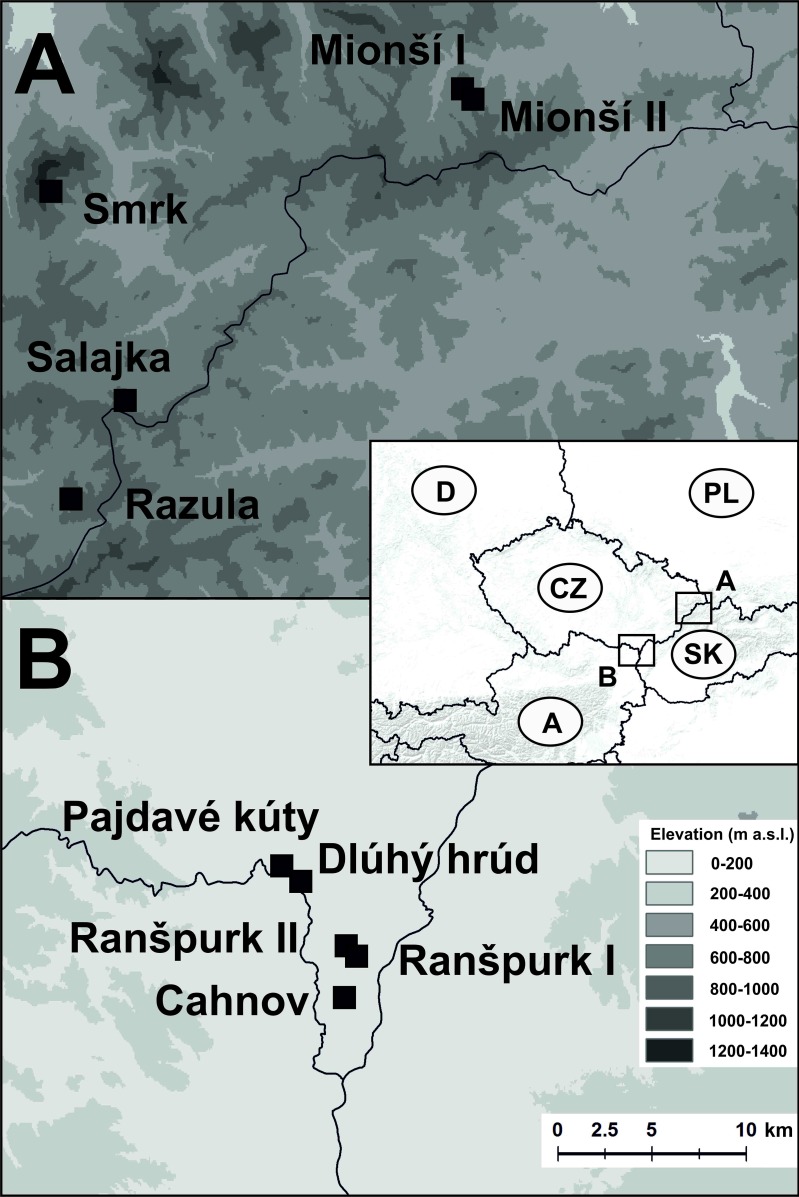
**Location of the study areas in Central Europe and positions of sampling sites** in the A) mountain forest and the B) lowland floodplain forest.

**Fig 2:**
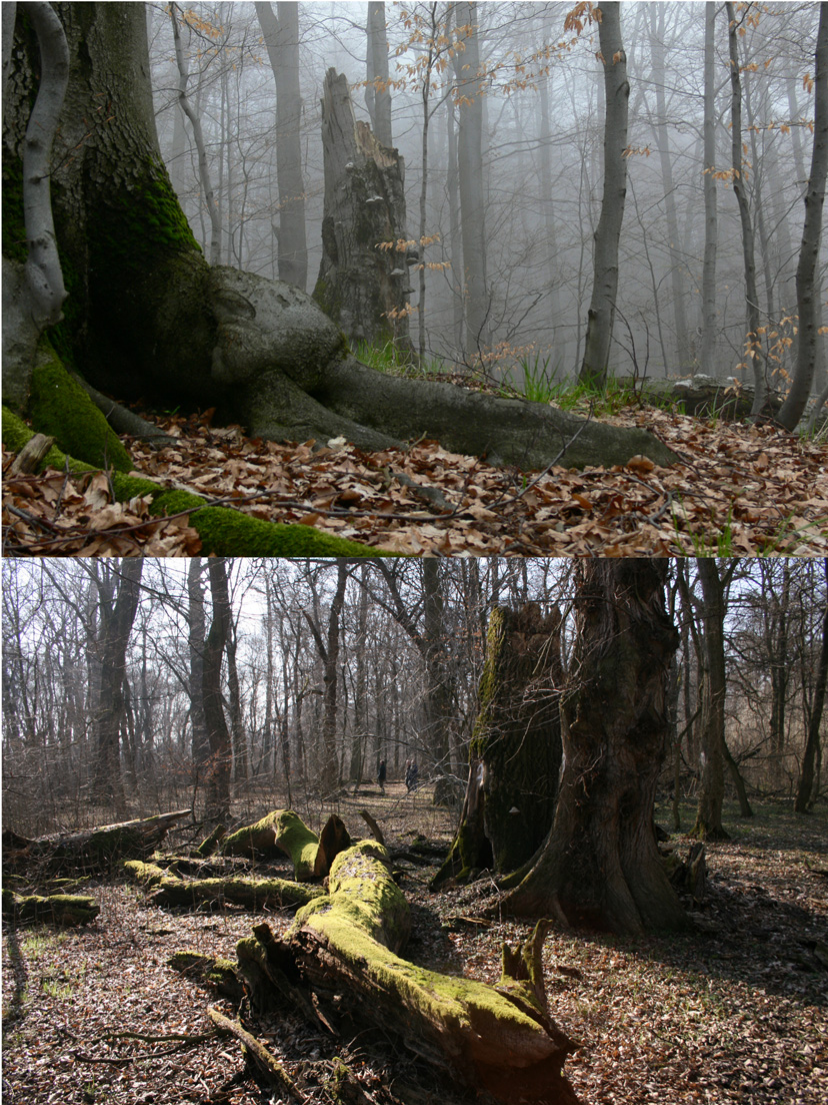
**Photo of the sampling sites** Mionší (top) in the montane forest and Ranšpurk in the lowland forest (below).

The sampling was performed in one lowland area and one mountain range in the Czech Republic. Both forest areas are characterized by diverse and near-to-natural tree species composition with a high volume of dead wood, many veteran trees and a rich, nearly complete saproxylic fauna. The lowland part of the study was conducted in alluvial woodlands along the lower Dyje (Thaya) and Morava (March) rivers in southern Moravia (48°37’- 53’ N, 16°36’- 17°05’ E; 150–153 m a.s.l., mean annual temperature 9°C, average annual precipitation 524 mm). The terrain was flat, the prevailing trees were pedunculate oak (*Quercus robur*), narrowleaf ash (*Fraxinus angustifolia*), hornbeam (*Carpinus betulus*), field maple (*Acer campestre*), interspersed with limes (*Tilia cordata*, *T*. *plat*y*phyllos*), European white elm (*Ulmus laevis*), poplars (*Populus alba*, *P*. *nigra*), and black alder (*Alnus glutinosa*). Historically, the forests were managed as coppice with standards or pasture woodland. These practices were abandoned 60–150 years ago [34]. Sampling was conducted in reserves and stands that had escaped the intensification of forestry, but nevertheless turned from oak-dominated sparse woodland to closed-canopy forest dominated by shade-tolerant species [35,36]. Five sites within the four largest remaining fragments of such stands in the area were included in the study (see below & Fig 1). The entire area is a regional biodiversity hotspot and important refuge of saproxylic fauna [36,37]. For an impression of the forest structure at the sampling sites see Fig 2.

The montane part of the study was conducted in the Moravian-Silesian Beskids (Beskydy)–a mountain range belonging to the Western Carpathians, situated in north-eastern Moravia, Czech Republic (49°10’- 39’ N, 17°59’- 18°44’ E, mean annual temperature 7°C, average annual precipitation 816 mm). The sampling sites had an elevational range of 715–1035 m a.s.l. (mean 814 m). Sampling was performed in reserves, historically partly managed as pasture forests, that have been left unmanaged for several decades [38]. The forest stands at the sites were dominated by European beech (*Fagus sylvatica*), interspersed with silver fir (*Abies alba*), Norway spruce (*Picea abies*), sycamore maple (*Acer pseudoplatanus*), European ash (*Fraxinus excelsior*) and Scotch elm (*Ulmus glabra*). The reserves are among the most important refuges of montane saproxylic biodiversity in the Czech Republic [39,40]. Five sites within four reserves were selected to match the situation in the lowland area (Fig 1). For an impression of the forest structure at the sampling sites see Fig 2.

The research in the lowland sites was conducted under the permit 8375/04-620/1377/04 issued by the Ministry of Environment of the Czech Republic. At the montane sites, the research was conducted under the exemption included in the Resolution of the Government of the Czech Republic No. 302. The research was performed on state owned land. The above permits grant access to the protected areas involved in the research, and allowed for sampling of insect species explicitly protected under national law.

### Sampling design and technique

The sampling design was identical in the lowlands and the mountains. Five sampling sites were selected in each of the two study areas (Fig 1). At each sampling site, three vertical transects were installed. Each vertical transect consisted of five traps exposed at 0.4, 1.2, 7, 14, and 21 meters above the ground (height at the middle of the interception panels). Hereafter the first two of these trap heights will be referred to as “understorey” and the other three as “canopy”. A total of 150 unbaited flight intercept traps were used, with 75 traps in lowland and 75 traps in montane forest. Sampling was carried out in 2007 in the lowland and in 2008 in the mountains. Due to the difference in the length of the vegetation season, traps at the lowland sites were operated from the end of March (after ceasing of inundation) until the end of September, whereas traps at the montane sites were operated from the end of April (ceasing of snow cover) until the end of September. The sampling thus covered virtually the whole period of beetle activity in both sampling areas.

In the lowland, vertical transects were installed at four sites, including Ranšpurk (two triplets, 48°40'42.946"N, 16°56'55.018"E and 48°40'40.446"N, 16°56'47.875"E), Cahnov (48°39'20.132"N, 16°56'26.013"E), Dlúhý hrúd (48°42'44.484"N, 16°54'15.171"E), and Pajdavé Kúty (48°43'4.638"N, 16°53'35.404"E). In the mountains, vertical transects were installed at four sites, including Mionší (two triplets, 49°32'15.947"N, 18°39'34.435"E and 49°32'4.330"N, 18°39'37.149"E), Salajka (49°24'8.243"N, 18°25'6.036"E), Razula (49°21'38.648"N, 18°22'43.441"E), and Smrk (49°29'38.484"N, 18°22'16.705"E).

The distance between the study areas was 150 km. The distances between transects within individual triplets (sites) was between 45 and 314 m (mean 103 m).

The flight intercept traps used were of the cross vane type (the two perpendicular transparent plastic panes were 50 cm high and 25 cm wide) with a roof, and a funnel connected to a collecting bottle with preservative (saturated salt solution with a drop of detergent to eliminate surface tension). The traps were emptied fortnightly. Relative cover of tree crowns (%) above the trap transect was recorded by a camera with fish-eye lens (16 mm focal length) and analysed using the software GapLightAnalyzer [41].

Beetles (Coleoptera) associated with dead wood (i.e. saproxylic and xylophagous ones) were used as the model group in order to avoid contamination of the dataset by species not associated with woodland habitatss. All trapped beetle individuals were sorted and identified to family level; saproxylic groups were identified to species level. Species identity was revised by experienced specialists. Staphylinidae were omitted from the dataset due to difficulties with their identification. This is a common approach, unlikely to affect our results [42,43]. Every saproxylic species was assigned to a feeding guild as either mycetophagous, xylophagous, zoophagous, or saprophagous based on the most authoritative information available [44]. All species in any way associated with fungi were considered as mycetophagous. Species were classified as threatened according to the Red List of threatened species in the Czech Republic Invertebrates [45]. Furthermore, species were classified as “primeval forest” species (stenotopic, and dispersal-limited species with close association to high quality forests habitats) according to [46]. The data are deposited in Dryad, a publicly accessible digital repository: http://dx.doi.org/10.5061/dryad.39k32

### Data Analysis

#### Species richness and distribution

For the purpose of the following analyses the data collected fortnightly were pooled per trap across the sampling period. To compare the overall species richness between mountains and lowland as well as among the sampled heights, the expected numbers of species with confidence intervals were computed using sample-based rarefaction using EstimateS 9.1.0 [47]. These analyses were conducted with the whole species data set for all samples of the two forest areas (N = 75) and for the individual sampling heights (N = 15). The total number of species was estimated using the classic Chao1 richness estimator with 100 runs for each of the two sampling areas [48]. Furthermore, the number of shared species between the pooled samples from each of the two elevations was estimated using the Chao shared species estimator [49].

#### Multivariate analyses

The relations among sample composition and explanatory variables were investigated using Redundancy Analysis (RDA), a linearly constrained ordination method that relates the species composition of samples to external predictors. RDA was chosen as a Detrended Correspondence Analysis conducted in a pre-analysis showed a gradient length of less than 3.0 SD units [50]. Separate ordinations were computed for the lowland and montane datasets. Trap height acted as the explanatory variable while sampling plot and canopy openness (as a surrogate for insolation) acted as covariables. All species with five or more individuals in the respective dataset were included in the analyses. Axes were tested with a Monte Carlo permutation test with 499 permutations. The same ordination was also used to carry out a variation partitioning analysis for the montane and lowland datasets. Sampling plot, trap height, and canopy openness were selected as explanatory variables in this analysis. Ordinations were carried out using Canoco 5 [51]. Traps represented samples characterised by captures of beetle species, and explanatory variables. The species abundance data acting as the response variable was log-transformed and centred by species. Trap height acted as a categorical variable. For constructing the ordination diagram scaling was focused on inter-sample distances and species scores were divided by standard deviations.

#### Dissimilarity patterns

Similarity between assemblages of the five sampling heights in terms of species composition was analysed by computing a Sorensen distance measure on all possible height pairings. For this purpose the data of each sampling height in a given elevation were pooled and then turned into presence-absence-data. Furthermore a measure of partitioning of the dissimilarity between sampling heights into its two components was applied: Dissimilarity reflects two phenomena–species turnover and nestedness. The first stands for replacement of species by others while the latter reflects species loss. Biotas with a smaller number of species that are only subsets of biotas at richer sites are considered nested [52]. To quantify the rate of change in assemblage composition along the vertical gradient, the Sorensen dissimilarity was standardised per 1 m of vertical distance between traps by dividing the values of dissimilarity between assemblages from two sampling heights by vertical distance (in m) between them. The Sorensen index (βsor), the Simpson dissimilarity index (βsim) describing spatial turnover and the nestedness-resultant dissimilarity (βnes) were computed with the *betapart* package [53] in R [54]. Species with less than five individuals were omitted from the analysis.

#### Feeding guilds

Species indicator values (IndVal) quantify the fidelity and specifity of species to groups of sites [55]. These values were computed for the montane and lowland assemblages to identify beetle species characteristic for the individual trap heights, using the *labdsv* package [56] in R [54]. Only species with five and more individuals were used for computing the IndVal and only those with an IndVal above 0.15 were selected as characteristic. A goodness-of-fit test was performed to test whether the characteristic species were evenly distributed across the heights. This test was applied to each of the four feeding guilds as well as to the sum of all characteristic species. Furthermore, a Chi-Square Test of probabilities was computed for each feeding guild to test if its distribution across the heights was identical with the distribution of all characteristic species, other guilds at the same elevation, or the same guild in the other elevation. The same test was also performed to test if the distribution of feeding guilds was different between the two elevations when all species were taken into account. The p-value was computed using a Monte Carlo simulation with 999 replicates, and Bonferroni correction was applied.

## Results

### Species richness and distribution

A total of 16,368 individuals of 512 saproxylic beetle species were caught (see species list in S1 Table). 7,429 beetle specimens were caught in the lowlands and 8,939 in the mountains. However, with 381 species trapped in the lowland and 236 in the mountains, the assemblage of the former was substantially richer. Only 105 species (21% of total species richness) were collected at both elevations; the number of shared species was estimated to be 169 (30%) using the Chao shared species estimator. The total number of species was estimated to be 463 (95% CI 428–519) in the lowlands and 319 (95% CI 276–380) in the mountains using the Chao1 species richness estimator. Of the species trapped in the lowland, 94 (25%) were red-listed and 67 (18%) were classified as “primeval forest” species; whereas of those trapped in the mountains, 32 (14%) were red-listed and 17 (7%) classified as “primeval forest” species.

**Fig 3:**
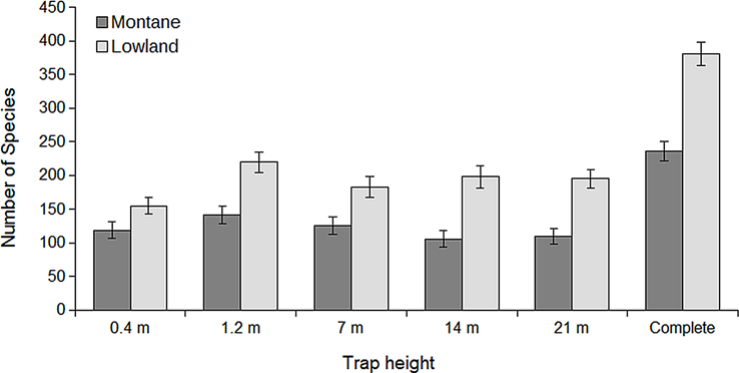
Species richness at the different height levels. Number of species (with 95% CI) of saproxylic beetles along the vertical gradient and overall species richness in the montane and lowland temperate forests as computed by sample-based rarefaction.

The number of species collected at a particular height was lower in the mountains for each of the sampled heights, and the difference in species richness was lowest near the ground and highest in the upper sampling heights. In both areas species richness peaked at 1.2 m. In the lowland, the assemblage at 0.4 m was the poorest, and there were no major differences in species richness among the three sampled heights in the canopy (7, 14, and 21 m; Fig 3). In the mountains, the higher canopy heights (14 and 21 m) were the poorest. There were more species collected at 0.4 and 7 m heights than higher in the canopy, but less than at 1.2 m height.

### Multivariate analyses

**Fig 4:**
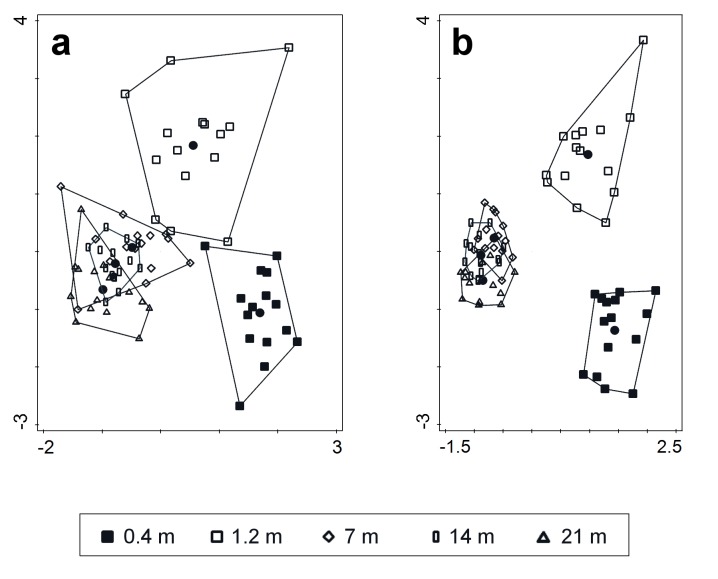
Redundancy Analysis ordination diagram of saproxylic beetle assemblages. The pooled assemblages from individual traps in the montane (a) and lowland (b) forests acted as samples (depicted), trap height acted as the explanatory variable and site and openness acted as covariables. The samples from the understorey (0.4 m, 1.2 m) are clearly separated from the canopy samples (7 m, 14 m, 21 m) along the 1st axis in both the montane (eigenvalue 1st axis = 0.1533) and the lowland datasets (eigenvalue 1st axis = 0.2162). The samples of the two understorey heights are separated along the 2nd axis in the mountains (eigenvalue 2nd axis = 0.0474) and lowlands (eigenvalue 2nd axis = 0.0505).

The Redundancy Analysis of the montane dataset revealed a clear difference between the species composition of the two understorey heights (0.4 and 1.2 m), as well as between the understorey and canopy heights, whereas the distinctions between the three canopy heights where minimal (eigenvalue 1^st^ axis = 0.1513, eigenvalue 2^nd^ axis = 0.0478; F = 15.5, p < 0.01) (Fig 4A). The same analysis of the lowland dataset (eigenvalue 1^st^ axis = 0.2036, eigenvalue 2^nd^ axis = 0.0448; F = 20.4, p < 0.01) yielded a very similar pattern (Fig 4B).

Variation partitioning showed that trap height accounted for 25.3% of the total variation (73.9% of the variation explained by all combined variables) in the lowland and for 19.6% (55.2% of explained variation) in the mountains, while the study site explained 7.3% (21.4% of explained variation) and 14% (39.5% of explained variation) in the lowland and in the mountains, respectively. Canopy openness explained < 2% of total variation (< 5% of explained variation) in both datasets. The three environmental variables and their combinations altogether explained 34.2% of the total variation in the lowlands and 35.5% in the mountains (Table 1). The permutation test on all ordination axes gave significant results for both montane (F = 6.1, p < 0.01) and lowland (F = 5.8, p < 0.01) data.

**Table 1:** Effect of environmental variables on composition of saproxylic beetle assemblages sampled along a vertical gradient in montane and lowland temperate forests. Computed by variation partitioning of Redundancy Analysis to show the amounts of variation explained by individual variables and their combinations.

	Montane		Lowland	
Environmental Variables	% of Explained Variation	% of Total Variation	% of Explained Variation	% of Total Variation
Study Site	39.5	14	21.4	7.3
Trap Height	55.2	19.6	73.9	25.3
% Canopy Openness	4.8	1.7	2	0.7
Study Site + Trap Height	- 4.4	- 1.6	- 3.9	- 1.3
Trap Height + % Canopy Openness	- 1.2	- 0.4	0	0
% Canopy Openness + Study Site	6.4	2.3	7.3	2.5
Study Site + Trap Height + % Canopy Openness	- 0.3	- 0.1	- 0.6	- 0.2
	**100**	**35.5**	**100**	**34.2**

### Species dissimilarity patterns

**Fig 5:**
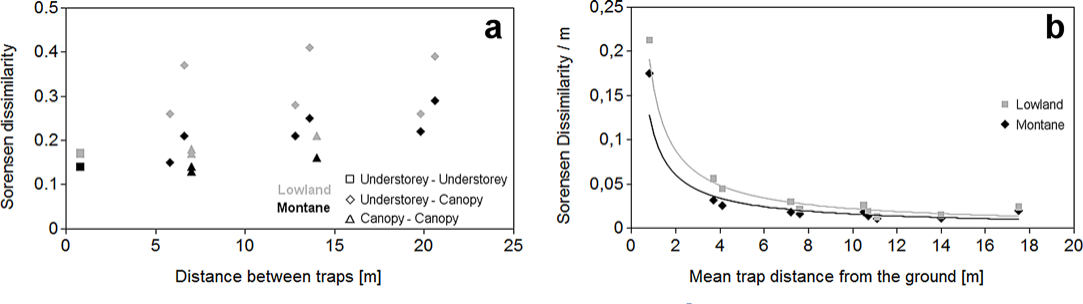
Dissimilarity of saproxylic beetle assemblages along a vertical forest gradient. (a) In both the lowland and montane forests Sorensen dissimilarity within the understorey (0.4 and 1.2 m heights above ground) and within the canopy (7, 14 and 21 m heights), was always lower than between samples from the two forest vertical strata. (b) The rate of change in assemblage composition decreased with distance from the ground along the vertical gradient. To standardize the Sorensen dissimilarity per 1 m of vertical distance between traps, the values of dissimilarity between assemblages from two trap heights were divided by vertical distance (in m) between them. These values are plotted against the mean height of the compared traps.

The Sorensen dissimilarity of beetle assemblages generally increased with the vertical distance between sampling heights in both datasets. It was, however, higher when comparing assemblages between canopy (7–21 m) and understorey (0.4–1.2 m) than within these layers regardless of the vertical distance (Fig 5A). The rate of change in beetle assemblage composition was highest near the ground and rapidly decreased with height in both elevations. The pattern was nearly identical for both elevations. When comparing assemblages of particular heights, the dissimilarity was always higher in the lowland than in the mountains (Fig 5B).

The amount of dissimilarity attributed to nestedness was generally low; it was mostly higher in the mountains than in the lowland. In the latter, the nestedness was highest for the two understorey assemblages, and then mostly decreased with the vertical distance between the respective heights. In the mountains, the nestedness was mostly high among the three canopy heights, while the differences in species composition between the two understorey heights were almost completely attributed to species turnover (Table 2).

**Table 2:** Results of Dissimilarity Partitioning showing what portion of the Sorensen Dissimilarity (β_SOR_) is accounted for by turnover (β_SIM_) and nestedness (β_NES_) for comparison of the beetle assemblages of all strata in montane (bold) and lowland (normal) forest. The dissimilarity was generally higher in the lowland and could mostly be attributed to turnover in both areas.

	0.4 m	1.2 m	7 m	14 m	21 m
**0.4 m**		β_SOR_ = 0.17	β_SOR_ = 0.37	β_SOR_ = 0.41	β_SOR_ = 0.39
		β_SIM_ = 0.04 (23%)	β_SIM_ = 0.31 (84%)	β_SIM_ = 0.35 (85%)	β_SIM_ = 0.32 (82%)
		β_NES_ = 0.13 (77%)	β_NES_ = 0.06 (16%)	β_NES_ = 0.06 (15%)	β_NES_ = 0.07 (18%)
**1.2 m**	**β**_**SOR**_ **= 0.14**		β_SOR_ = 0.26	β_SOR_ = 0.28	β_SOR_ = 0.26
	**β**_**SIM**_ **= 0.11 (79%)**		β_SIM_ = 0.22 (85%)	β_SIM_ = 0.24 (86%)	β_SIM_ = 0.23 (88%)
	**β**_**NES**_ **= 0.03 (21%)**		β_NES_ = 0.04 (15%)	β_NES_ = 0.04 (14%)	β_NES_ = 0.03 (12%)
**7 m**	**β**_**SOR**_ **= 0.21**	**β**_**SOR**_ **= 0.15**		β_SOR_ = 0.18	β_SOR_ = 0.21
	**β**_**SIM**_ **= 0.19 (90%)**	**β**_**SIM**_ **= 0.13 (87%)**		β_SIM_ = 0.17 (94%)	β_SIM_ = 0.20 (95%)
	**β**_**NES**_ **= 0.02 (10%)**	**β**_**NES**_ **= 0.02 (13%)**		β_NES_ = 0.01 (6%)	β_NES_ = 0.01 (5%)
**14 m**	**β**_**SOR**_ **= 0.25**	**β**_**SOR**_ **= 0.21**	**β**_**SOR**_ **= 0.13**		β_SOR_ = 0.17
	**β**_**SIM**_ **= 0.23 (92%)**	**β**_**SIM**_ **= 0.16 (76%)**	**β**_**SIM**_ **= 0.09 (69%)**		β_SIM_ = 0.16 (94%)
	**β**_**NES**_ **= 0.02 (8%)**	**β**_**NES**_ **= 0.05 (24%)**	**β**_**NES**_ **= 0.04 (31%)**		β_NES_ = 0.01 (6%)
**21 m**	**β**_**SOR**_ **= 0,29**	**β**_**SOR**_ **= 0.22**	**β**_**SOR**_ **= 0.16**	**β**_**SOR**_ **= 0.14**	
	**β**_**SIM**_ **= 0.25 (86%)**	**β**_**SIM**_ **= 0.15 (68%)**	**β**_**SIM**_ **= 0.10 (60%)**	**β**_**SIM**_ **= 0.12 (86%)**	
	**β**_**NES**_ **= 0.04 (14%)**	**β**_**NES**_ **= 0.07 (32%)**	**β**_**NES**_ **= 0.06 (40%)**	**β**_**NES**_ **= 0.02 (14%)**	

### Feeding guilds

**Fig 6:**
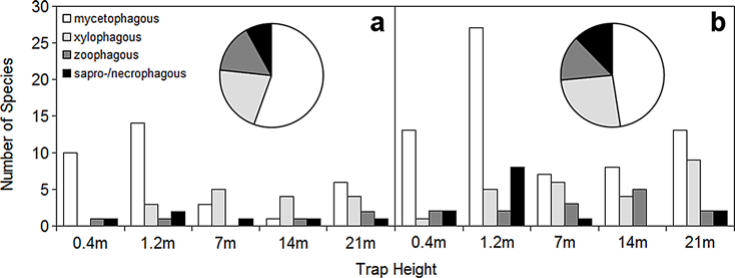
**Guild structure of saproxylic beetle assemblages in montane (a) and lowland (b) temperate forests (pie charts), and along a vertical gradient in both forest types (barplots).** The overall guild structure includes all recorded species. The barplots depict guild identity of species identified by indicator value analysis as characteristic for the given sampling height.

Of the height-characteristic species identified by IndVal in the mountains, most of the mycetophages were associated with the understorey (0.4 & 1.2 m), whereas the xylophages were mostly associated with the canopy (7, 14 & 21 m). In the lowland the mycetophages were associated with the understorey, but also with the canopy at 21 m. The xylophages peaked at 21 m, followed by 7 m (see Fig 6). The goodness-of-fit test on all the characteristic species showed that in the mountains only the mycetophages were significantly unevenly distributed among the sampled heights (χ^2^ = 16.294, p < 0.01), while the result for all characteristic species was marginally insignificant (χ^2^ = 8.098, p = 0.086). The Chi-Square Test of probabilities showed that in the mountains the distribution of xylophages differed from that of all the characteristic species (χ^2^ = 9.712, p < 0.05) and that of the mycetophages (χ^2^ = 63.523, p < 0.01). The distribution of the mycetophages also differed from that of the predators (χ^2^ = 14.162, p < 0.01). In the lowland, neither all the characteristic species (χ^2^ = 19.25, p < 0.001) nor the mycetophages (χ^2^ = 18.765, p < 0.001) and saprophages (χ^2^ = 15.076, p < 0.01) were evenly distributed among the sampled heights. The distribution of the mycetophages differed from that of the xylophages (χ^2^ = 63.523, p < 0.01) and the predators (χ^2^ = 47.836, p < 0.01). Likewise, the distribution of the saprophages differed from that of the xylophages (χ^2^ = 20.482, p < 0.01) and the predators (χ^2^ = 26.128, p < 0.01). Furthermore, the distributions of the xylophages and predators differed marginally (χ^2^ = 13.472, p < 0.05).

None of the guilds, however, showed a significantly different distribution between the mountain and lowland forest. The overall distribution of feeding guilds differed only marginally (χ^2^ = 8.979, p < 0.05) between the two sampling areas. The overall representation of the feeding guilds was rather similar between mountains and lowland: In both areas mycetophages made up the largest share of beetle species. The share of predators was roughly the same in both elevations, while that of xylophages and saprophages was slightly higher in the lowlands.

## Discussion

### Study outcome and limitations

Our results bring novel information on diversity and guild structure of saproxylic beetle assemblages along fine-scale vertical gradient in temperate lowland and montane forests. Sampling only two forest areas partly limits the validity of our observations. On the other hand, both of the sampling areas are diversity hot-spots of saproxylic fauna and refuges of the last populations of many highly endangered saproxylic species in the wider region [39,40,36]. Their fauna is thus representative of the habitat. It would be difficult to find other suitable lowland sites due to the high human pressure on lowland forests of Central Europe [57]; inclusion of impoverished sites would lead rather to underestimation of lowland diversity than to more precise results. Further, the higher amount of variability explained by the sampling site in the mountains (reflecting the distances among and the wider altitudinal range of the sites) shows that the sampling covered higher habitat diversity in the mountains, thus potentially leading rather to overestimation than underestimation of beetle diversity there.

The sampling sites spread over many square kilometres within each sampling area, the sampled areas are representative of habitat types common in Europe and elsewhere, but biologically as diverse as possible. The sampling was intensive and its design followed an identical protocol in both sampling areas. We therefore believe the resulting data are comparable and the results are relevant to the wider region.

### Diversity and conservation value of lowland and montane assemblages

The lowland forest accommodated substantially (61%) more species of saproxylic beetles than the montane forest. The turnover of saproxylic beetles between lowland and mountains was substantial, as only about 21% of all sampled species were estimated to occur at both elevations. The lowlands thus hosted a much larger share of species present also in the mountains, than vice versa. Further, the lowland hosted a substantially higher portion of red-listed [45] and “primeval forest” species [46].

Although mountains are an important refuge, our results underline the high importance of lowland forests for conservation of saproxylic biodiversity. Although based on the sampling of only two forest areas (*see above*), our results are fully in line with the findings of other studies (e.g. [58]) and clearly indicate that the conservation of saproxylic beetles in Europe would benefit from focusing more on lowlands. This is, however, not meant to downplay the value of the montane habitats for the preservation of saproxylic beetles since both forests hosted rather distinct communities.

### Saproxylic beetle diversity along the vertical gradient

In both study areas the saproxylic beetle fauna displayed clear signs of stratification along the vertical gradient. There were considerable differences in the assemblage composition between the heights sampled in the canopy (7, 14, 21 m) and the understorey (0.4, 1.2 m). This is in accordance with other studies reporting stratification of saproxylic beetles between canopy and understorey of temperate forests (e.g. [14,30,31,59,32]).

Our results also show differences within both the canopy and the understorey strata. While the differences, as shown by the multivariate analysis, among the three heights sampled in the canopy were rather small, the differences between the two heights sampled in the understorey were substantial at both elevations. The dissimilarity was nearly identical between the two sampling heights in the understorey (0.8 m vertical distance) as among the three sampling heights within the canopy (vertical distance 7 and 14 m). It was also higher when comparing samples between understorey and canopy than within them. In comparison to dissimilarity measures, the multivariate analyses showed even more difference between the two understorey heights. This is, most likely, owing to the fact that the former is based on species presence/absence data, while the latter accounts also for abundances.

We may thus conclude that there is neither a sudden change in assemblage composition along the vertical gradient, nor is there a clear boundary between understorey and canopy in the sampled forests. The rate of change in assemblage composition, however, rapidly decreases with the distance from the ground. The high diversity of dead wood microhabitats and generally high availability of dead wood close to the ground in combination with the rapid change of microclimate near the forest floor are likely the reasons for the observed pattern. Their effects on beetle assemblages are gradually fading somewhere between 1.2 and 7 m above ground in the habitats studied here. This indicates that despite a gradual change, the transition between canopy and understorey occurs somewhere between these two heights in a temperate forest. The high diversity at the 1.2 m height could, perhaps, be partly explained by the overlap between canopy and understorey fauna. Since predation pressure is high on the ground [60] and herbs and shrubs are concentrated near the ground, the 1.2 m height level might also represent a relatively enemy-free and obstacle-free space frequented even by species exploiting resources found below this height [27].

Although we found high accordance in vertical stratification of saproxylic beetles between the two elevations, there were also notable differences. Firstly, the multivariate analyses as well as the dissimilarity partitioning indicated that the beetle assemblages were more stratified in the lowland. Secondly, the patterns of species richness along the vertical gradient differed between the two elevations. While the number of species declined with height in the canopy of the montane forest, there was no difference among the three sampled heights in the canopy of lowland forest, and the data even suggested an opposite pattern. All of this might be explained by the more complex vertical structure of the lowland forest, which shows more specific tree layers and therefore a higher variability of habitats [61]. In the same way, the stronger vertical stratification in tropical forests in comparison temperate forests has been attributed to their more complex vertical structure [11]. Further, the lowest height was the poorest in the lowland, but not in the mountains. The denser undergrowth and the occasional floods at the lowland sites might be responsible for the low beetle numbers at the lowest sampling height.

Many studies concerning vertical stratification are focused on the question of whether the canopy is richer than understorey, or vice versa. Our results from the lowland demonstrate that the outcome of such comparisons may give contrasting results depending on the exact height sampled in the understorey. This, together with the effect of local environmental conditions on vertical stratification of insects [62,14,63,59] offers another explanation for the inconsistent and often contrasting outcomes of studies dealing with vertical stratification of insects in forests (cf. [64,65,66,31]).

We did not sample the upper canopy, thus missing a potentially important part of the stratum. However, the similarity of the assemblages across the three canopy heights sampled in this study, together with the results of [14], makes it unlikely that the addition of another sampling height in the canopy would have caused a substantial change of the study outcome. The documented within-strata differences are highly similar to the results of a study of comparable design, that showed a significant decrease of species similarity along their vertical gradient. This illustrates that sampling on a finer scale is indeed crucial for a better understanding of insect vertical distribution in forests [11]. Our results show that the knowledge of vertical stratification would benefit from finer sampling of those parts of the vertical forest gradient where the change of abiotic factors is most pronounced, i.e. near the ground and, possibly, also in the upper canopy.

### Distribution of feeding guilds

Despite the differences in species richness and assemblage composition, guild structure was surprisingly similar between lowland and mountains. The largest share of assemblages consisted of the mycetophages, followed by the xylophages and the zoophages. The saprophages constituted the lowest share in both elevations. The main difference was the higher share of xylophages in the lowland compared to the mountains. This is explainable by the fact that lowlands are generally warmer and drier. The climate thus likely favours the xylophages and allows them to exploit a larger share of the available resource [67]. The cooler and more humid montane climate is likely to favour wood-decaying fungi, as also suggested by the higher diversity of fungal communities found at higher elevations [68,2,3]. This is also supported by higher diversity of fungi-associated beetles in moist forests [69].

The distribution of feeding guilds along the vertical gradient was mostly similar between lowland and mountains. Mycetophages were mostly concentrated in the understorey, probably owing to the fact that higher humidity near the ground provides better conditions for fungi [70,32]. While there were almost no fungi feeders characteristic of the canopy in the montane forest, such species showed a notable presence in the lowland. Predators did not show a clear preference for any specific height or stratum, a finding similar to the results a recent study [32]. The saprophages were concentrated in the understorey in the lowland but not in the montane forest. Despite the above-mentioned differences the patterns of the guild stratification are rather similar in the two forest types. The lack of a clear trend in guilds of lower species richness (predators and saprophages) may reflect an insufficient amount of indicator species rather than reality.

### Conclusions

We conclude that temperate lowland forests hosts a substantially more diverse and threatened saproxylic beetle fauna than montane forests. Therefore, while conservation efforts should concern both types of habitats, the attention on lowland forests must be increased to preserve saproxylic species richness in Central Europe. Saproxylic beetles are stratified not only between the understorey and the canopy of temperate forests, but also within the understorey. The rate of change of the beetle community along vertical gradients decreases with distance from the ground. The comparisons of beetle richness between canopy and understorey may thus give contrasting results depending on the exact height sampled in the latter. Despite the fact that species composition differed substantially between montane and lowland forests, most patterns of feeding guild representation and vertical distribution were rather similar in the two forest types.

## Supporting Information

S1 Table List of sampled beetle species. Information on Family, number of specimen in the mountain and lowland forest, sparoxylic status (**1**: obligatory; **2**: facultative; **3**: potentially), trophic guild (**m**: mycetophagous; **n**: necrophagous; **p**: phytopagous; **s**: saprophagous; **x**: xylophagous; **z**: zoophagous), red list status (**CR**: critically endangered; **EN**: endangered; **VU**: vulnerable; **NT**: near threatened) and status as indicator species (Schmidl & Bussler 2004).
(DOC)
