# Supplementary material for: Fine-Scale Vertical Stratification and Guild Composition of Saproxylic Beetles in Lowland and Montane Forests: Similar Patterns despite Low Faunal Overlap
Source: PLoS One. 2016 Mar 15;11(3):e0149506. doi: 10.1371/journal.pone.0149506 (PMC4792385; doi:10.1371/journal.pone.0149506)
Supplement: S1 Table — Information on Family, number of specimen in the mountain and lowland forest, sparoxylic status (1: obligatory; 2: facultative; 3: potentially), trophic guild (m: mycetophagous; n: necrophagous; p: phytopagous; s: saprophagous; x: xylophagous; z: zoophagous), red list status (CR: critically endangered; EN: endangered; VU: vulnerable; NT: near threatened) and status as indicator species (Schmidl & Bussler 2004). (DOC) [file pone.0149506.s001.doc]

**S1 Table: List of sampled beetle species.** Information on Family, number of specimen in the mountain and lowland forest, sparoxylic status (**1**: obligatory; **2**: facultative; **3**: potentially), trophic guild (**m**: mycetophagous; **n**: necrophagous; **p**: phytopagous; **s**: saprophagous; **x**: xylophagous; **z**: zoophagous), red list status (**CR**: critically endangered; **EN**: endangered; **VU**: vulnerable; **NT**: near threatened) and status as indicator species (Schmidl & Bussler 2004).

| Species | Author | Family | Specimen Montane | Specimen Lowland | Saproxylic Status | Trophic Guild | Red List Status | “Primeval Forest“ Species |
| --- | --- | --- | --- | --- | --- | --- | --- | --- |
| *Euglenes oculatus* | (Paykull, 1798) | Aderidae | 0 | 4 | 1 | s | - | no |
| *Phytobaenus amabilis* | R.F. Sahlberg, 1834 | Aderidae | 0 | 3 | 1 | s | EN | no |
| *Pseudanidorus pentatomus* | (C.G. Thomson, 1864) | Aderidae | 1 | 0 | 1 | s | - | yes |
| *Choragus sheppardi* | Kirby, 1819 | Anthribidae | 0 | 1 | 1 | m | - | no |
| *Dissoleucas niveirostris* | (Fabricius, 1798) | Anthribidae | 0 | 1 | 1 | m | - | no |
| *Enedreytes sepicola* | (Scopoli, 1763) | Anthribidae | 0 | 1 | 1 | m | NT | no |
| *Platyrhinus resinosus* | (Linnaeus, 1758) | Anthribidae | 0 | 4 | 1 | m | NT | no |
| *Platystomos albinus* | (Fabricius, 1792) | Anthribidae | 0 | 2 | 1 | m | - | no |
| *Rhaphitropis marchica* | (Herbst, 1792) | Anthribidae | 0 | 1 | 1 | m | - | no |
| *Diplocoelus fagi* | Chevrolat, 1837 | Biphyllidae | 0 | 1 | 1 | m | - | no |
| *Teredus cylindricus* | (A. G. Olivier, 1790) | Bothrideridae | 0 | 1 | 1 | z | VU | yes |
| *Agrilus biguttatus* | Fabricius, 1777 | Buprestidae | 0 | 10 | 1 | x | - | no |
| *Agrilus convexicollis* | L. Redtenbacher, 1849 | Buprestidae | 0 | 35 | 1 | x | VU | no |
| *Agrilus cuprescens* | (Ménétriés, 1832) | Buprestidae | 0 | 1 | 1 | x | - | no |
| *Agrilus graminis* | Kiesenwetter, 1857 | Buprestidae | 0 | 5 | 1 | x | - | no |
| *Agrilus hastulifer* | Ratzeburg, 1837 | Buprestidae | 0 | 2 | 1 | x | EN | yes |
| *Agrilus laticornis* | (Illiger, 1803) | Buprestidae | 0 | 44 | 1 | x | - | no |
| *Agrilus obscuricollis* | Kiesenwetter, 1857 | Buprestidae | 0 | 1 | 1 | x | - | no |
| *Agrilus olivicolor* | Kiesenwetter, 1857 | Buprestidae | 0 | 26 | 1 | x | NT | no |
| *Agrilus sulcicollis* | Lacordaire, 1835 | Buprestidae | 0 | 6 | 1 | x | - | no |
| *Agrilus suvorovi* | Obenberger, 1935 | Buprestidae | 0 | 2 | 1 | x | NT | no |
| *Agrilus viridis* | Linnaeus, 1758 | Buprestidae | 4 | 0 | 1 | x | - | no |
| *Anthaxia podolica* | Mannerheim, 1837 | Buprestidae | 0 | 1 | 1 | x | VU | yes |
| *Anthaxia semicuprea* | Küster, 1851 | Buprestidae | 0 | 2 | 1 | x | EN | yes |
| *Anthaxia senicula* | Schrank, 1789 | Buprestidae | 0 | 1 | 1 | x | - | no |
| *Eurythyrea quercus* | (Herbst, 1784) | Buprestidae | 0 | 1 | 1 | x | CR | yes |
| *Malthinus facialis* | C.G. Thomson, 1864 | Cantharidae | 1 | 0 | 1 | z | VU | no |
| *Malthinus flaveolus* | (Herbst, 1786) | Cantharidae | 6 | 0 | 2 | z | - | no |
| *Malthinus frontalis* | (Marsham, 1802) | Cantharidae | 1 | 0 | 2 | z | - | no |
| *Malthodes fuscus* | (Waltl, 1838) | Cantharidae | 1 | 0 | 2 | z | - | no |
| *Malthodes guttifer* | Kiesenwetter, 1852 | Cantharidae | 2 | 0 | 2 | z | - | no |
| *Malthodes hexacanthus* | Kiesenwetter, 1852 | Cantharidae | 2 | 0 | 2 | z | - | no |
| *Malthodes lobatus* | Kiesenwetter, 1852 | Cantharidae | 1 | 0 | 2 | z | VU | no |
| *Malthodes marginatus* | (Latreille, 1806) | Cantharidae | 1 | 0 | 2 | z | - | no |
| *Malthodes pumilus* | (Brébisson, 1835) | Cantharidae | 2 | 0 | 2 | z | - | no |
| *Akimerus schaefferi* | (Laicharting, 1784) | Cerambycidae | 0 | 1 | 1 | x | CR | yes |
| *Anaglyptus mysticus* | (Linnaeus, 1758) | Cerambycidae | 0 | 3 | 1 | x | - | no |
| *Anastrangalia dubia* | (Scopoli, 1763) | Cerambycidae | 1 | 0 | 1 | x | - | no |
| *Cerambyx cerdo* | Linnaeus, 1758 | Cerambycidae | 0 | 1 | 1 | x | EN | yes |
| *Clytus arietis* | (Linnaeus, 1758) | Cerambycidae | 0 | 2 | 1 | x | - | no |
| *Evodinus clathratus* | (Fabricius, 1792) | Cerambycidae | 1 | 0 | 1 | x | - | no |
| *Exocentrus adspersus* | Mulsant, 1846 | Cerambycidae | 0 | 1 | 1 | x | - | no |
| *Exocentrus lusitanus* | (Linnaeus, 1767) | Cerambycidae | 0 | 2 | 1 | x | - | no |
| *Glaphyra umbellatarum* | (Schreber, 1759) | Cerambycidae | 0 | 2 | 1 | x | - | no |
| *Grammoptera ruficornis* | (Fabricius, 1781) | Cerambycidae | 0 | 1 | 1 | x | - | no |
| *Grammoptera ustulata* | (Schaller, 1783) | Cerambycidae | 0 | 2 | 1 | x | - | no |
| *Leioderes kollari* | L. Redtenbacher, 1849 | Cerambycidae | 0 | 3 | 1 | x | NT | no |
| *Leiopus nebulosus* | (Linnaeus, 1758) | Cerambycidae | 3 | 3 | 1 | x | - | no |
| *Mesosa nebulosa* | (Fabricius, 1781) | Cerambycidae | 0 | 3 | 1 | x | - | no |
| *Molorchus minor* | (Linnaeus, 1758) | Cerambycidae | 2 | 0 | 1 | x | - | no |
| *Obrium brunneum* | (Fabricius, 1792) | Cerambycidae | 1 | 0 | 1 | x | - | no |
| *Oxymirus cursor* | Linnaeus, 1758 | Cerambycidae | 5 | 0 | 1 | x | - | no |
| *Phymatodes alni* | (Linnaeus, 1767) | Cerambycidae | 0 | 1 | 1 | x | - | no |
| *Phymatodes rufipes* | (Fabricius, 1777) | Cerambycidae | 0 | 2 | 1 | x | - | no |
| *Plagionotus detritus* | (Linnaeus, 1758) | Cerambycidae | 0 | 1 | 1 | x | - | yes |
| *Pogonocherus hispidus* | (Linnaeus, 1758) | Cerambycidae | 0 | 2 | 1 | x | - | no |
| *Prionus coriarius* | (Linnaeus, 1758) | Cerambycidae | 0 | 3 | 1 | x | - | no |
| *Pyrrhidium sanguineum* | (Linnaeus, 1758) | Cerambycidae | 1 | 0 | 1 | x | - | no |
| *Rhagium mordax* | (DeGeer, 1775) | Cerambycidae | 3 | 6 | 1 | x | - | no |
| *Rhagium sycophanta* | (Schrank, 1781) | Cerambycidae | 0 | 4 | 1 | x | NT | yes |
| *Rosalia alpina* | (Linnaeus, 1758) | Cerambycidae | 0 | 1 | 1 | x | CR | yes |
| *Saperda punctata* | (Linnaeus, 1767) | Cerambycidae | 0 | 1 | 1 | x | EN | no |
| *Stenocorus meridianus* | (Linnaeus, 1758) | Cerambycidae | 0 | 11 | 1 | x | - | no |
| *Stictoleptura scutellata* | (Fabricius, 1781) | Cerambycidae | 0 | 1 | 1 | x | - | yes |
| *Tetropium castaneum* | (Linnaeus, 1758) | Cerambycidae | 3 | 0 | 1 | x | - | no |
| *Tetrops praeustus* | (Linnaeus, 1758) | Cerambycidae | 0 | 1 | 1 | x | - | no |
| *Cerylon deplanatum* | Gyllenhal, 1827 | Cerylonidae | 3 | 1 | 1 | s | EN | no |
| *Cerylon fagi* | C. N. F. Brisout de Barneville, 1867 | Cerylonidae | 3 | 0 | 1 | s | - | no |
| *Cerylon ferrugineum* | Stephens, 1830 | Cerylonidae | 29 | 135 | 1 | s | - | no |
| *Cerylon histeroides* | (Fabricius, 1792) | Cerylonidae | 11 | 267 | 1 | s | - | no |
| *Cis boleti* | (Scopoli, 1763) | Ciidae | 19 | 358 | 1 | m | - | no |
| *Cis castaneus* | (Herbst, 1793) | Ciidae | 8 | 0 | 1 | m | - | no |
| *Cis comptus* | Gyllenhal, 1827 | Ciidae | 1 | 33 | 1 | m | - | no |
| *Cis dentatus* | Mellié, 1848 | Ciidae | 11 | 0 | 1 | m | VU | no |
| *Cis fagi* | Waltl, 1839 | Ciidae | 6 | 0 | 1 | m | - | no |
| *Cis festivus* | (Panzer, 1793) | Ciidae | 3 | 15 | 1 | m | - | no |
| *Cis fusciclavis* | Nyholm, 1953 | Ciidae | 0 | 2 | 1 | m | - | no |
| *Cis glabratus* | Mellié, 1848 | Ciidae | 15 | 0 | 1 | m | - | no |
| *Cis jacquemartii* | Mellié, 1848 | Ciidae | 3 | 0 | 1 | m | - | no |
| *Cis lineatocribratus* | Mellié, 1848 | Ciidae | 1 | 0 | 1 | m | VU | no |
| *Cis micans* | (Fabricius, 1792) | Ciidae | 13 | 24 | 1 | m | - | no |
| *Cis punctulatus* | Gyllenhal, 1827 | Ciidae | 1 | 0 | 1 | m | - | no |
| *Cis pygmaeus* | (Marsham, 1802) | Ciidae | 1 | 14 | 1 | m | - | no |
| *Cis rugulosus* | Mellié, 1848 | Ciidae | 65 | 2 | 1 | m | - | no |
| *Cis vestitus* | (Mellié, 1848) | Ciidae | 4 | 11 | 1 | m | - | no |
| *Cis villosulus* | Marsham, 1802 | Ciidae | 4 | 7 | 1 | m | - | no |
| *Ennearthron cornutum* | (Gyllenhal, 1827) | Ciidae | 18 | 8 | 1 | m | - | no |
| *Octotemnus glabriculus* | (Gyllenhal, 1827) | Ciidae | 1 | 4 | 1 | m | - | no |
| *Orthocis alni* | (Gyllenhal, 1813) | Ciidae | 1 | 21 | 1 | m | - | no |
| *Orthocis lucasi* | (Abeille de Perrin, 1874) | Ciidae | 1 | 8 | 1 | m | - | no |
| *Rhopalodontus baudueri* | Abeille de Perrin, 1874 | Ciidae | 1 | 0 | 1 | m | - | no |
| *Rhopalodontus novorossicus* | Reitter, 1902 | Ciidae | 0 | 5 | 1 | m | CR | yes |
| *Rhopalodontus perforatus* | (Gyllenhal, 1813) | Ciidae | 2 | 0 | 1 | m | - | no |
| *Sulcacis fronticornis* | (Panzer, 1809) | Ciidae | 0 | 5 | 1 | m | - | no |
| *Sulcacis nitidus* | (Fabricius, 1792) | Ciidae | 0 | 6 | 1 | m | - | no |
| *Opilo mollis* | (Linnaeus, 1758) | Cleridae | 0 | 2 | 1 | z | - | no |
| *Opilo pallidus* | (A. G. Olivier, 1795) | Cleridae | 0 | 1 | 1 | z | VU | yes |
| *Thanasimus formicarius* | (Linnaeus, 1758) | Cleridae | 4 | 9 | 1 | z | - | no |
| *Tilloidea unifasciata* | (Fabricius, 1787) | Cleridae | 0 | 6 | 1 | z | - | yes |
| *Tillus elongatus* | (Linnaeus, 1758) | Cleridae | 11 | 6 | 1 | z | - | no |
| *Arthrolips nana* | (Mulsant & Rey, 1861) | Corylophidae | 0 | 19 | 1 | m | - | no |
| *Arthrolips obscura* | (C. R. Sahlberg, 1833) | Corylophidae | 0 | 10 | 3 | m | - | no |
| *Clypastraea brunnea* | C. N. F. Brisout de Barneville, 1863 | Corylophidae | 0 | 8 | 3 | m | - | no |
| *Clypastraea reitteri* | Bowestead, 1999 | Corylophidae | 0 | 8 | 3 | m | - | no |
| *Orthoperus atomus* | (Gyllenhal, 1808) | Corylophidae | 0 | 20 | 3 | m | - | no |
| *Orthoperus brunnipes* | (Gyllenhal, 1808) | Corylophidae | 0 | 21 | 3 | m | - | no |
| *Orthoperus corticalis* | (L. Redtenbacher, 1849) | Corylophidae | 0 | 35 | 3 | m | - | no |
| *Sericoderus lateralis* | (Gyllenhal, 1827) | Corylophidae | 0 | 26 | 3 | m | - | no |
| *Atomaria affinis* | R.F. Sahlberg, 1834 | Cryptophagidae | 9 | 16 | 2 | m | - | no |
| *Atomaria alpina* | Heer, 1841 | Cryptophagidae | 0 | 3 | 2 | m | - | no |
| *Atomaria analis* | Erichson, 1846 | Cryptophagidae | 0 | 3 | 2 | m | - | no |
| *Atomaria atricapilla* | Stephens, 1830 | Cryptophagidae | 0 | 15 | 3 | m | - | no |
| *Atomaria badia* | Erichson, 1846 | Cryptophagidae | 33 | 0 | 1 | m | - | no |
| *Atomaria diluta* | Erichson, 1846 | Cryptophagidae | 0 | 2 | 1 | m | - | no |
| *Atomaria elongatula* | Erichson, 1846 | Cryptophagidae | 2 | 338 | 2 | m | - | no |
| *Atomaria fimetaria* | (Herbst, 1793) | Cryptophagidae | 0 | 1 | 3 | m | - | no |
| *Atomaria fuscata* | (Schönherr, 1808) | Cryptophagidae | 0 | 13 | 3 | m | - | no |
| *Atomaria nigrirostris* | Stephens, 1830 | Cryptophagidae | 0 | 13 | 3 | m | - | no |
| *Atomaria ornata* | Heer, 1841 | Cryptophagidae | 7 | 0 | 1 | m | - | no |
| *Atomaria puncticollis* | C. G. Thomson, 1868 | Cryptophagidae | 2 | 0 | 2 | m | - | no |
| *Atomaria slavonica* | C. Johnson, 1971 | Cryptophagidae | 0 | 1 | 3 | m | - | no |
| *Atomaria turgida* | Erichson, 1846 | Cryptophagidae | 2 | 1 | 2 | m | - | no |
| *Atomaria vespertina* | Mäklin, 1853 | Cryptophagidae | 0 | 2 | 2 | m | - | no |
| *Caenoscelis ferruginea* | (C. R. Sahlberg, 1820) | Cryptophagidae | 0 | 1 | 2 | m | - | no |
| *Cryptophagus badius* | Sturm, 1845 | Cryptophagidae | 11 | 0 | 1 | m | - | no |
| *Cryptophagus confusus* | Bruce, 1934 | Cryptophagidae | 0 | 2 | 2 | m | - | no |
| *Cryptophagus cylindrellus* | Johnson 2007 | Cryptophagidae | 3 | 0 | 2 | m | - | no |
| *Cryptophagus dentatus* | (Herbst, 1793) | Cryptophagidae | 63 | 16 | 2 | m | - | no |
| *Cryptophagus denticulatus* | Heer, 1841 | Cryptophagidae | 3 | 2 | 2 | m | - | no |
| *Cryptophagus fuscicornis* | Sturm, 1845 | Cryptophagidae | 3 | 107 | 2 | m | - | no |
| *Cryptophagus labilis* | Erichson, 1846 | Cryptophagidae | 3 | 0 | 1 | m | - | no |
| *Cryptophagus lapponicus* | Gyllenhal, 1827 | Cryptophagidae | 3 | 0 | 2 | m | - | no |
| *Cryptophagus pallidus* | Sturm, 1845 | Cryptophagidae | 0 | 2 | 2 | m | - | no |
| *Cryptophagus pubescens* | Sturm, 1845 | Cryptophagidae | 1 | 5 | 2 | m | - | no |
| *Cryptophagus punctipennis* | C. N. F. Brisout de Barneville, 1863 | Cryptophagidae | 3 | 1 | 3 | m | - | no |
| *Cryptophagus quercinus* | Kraatz, 1852 | Cryptophagidae | 2 | 0 | 2 | m | - | no |
| *Cryptophagus reflexus* | Rey, 1982 | Cryptophagidae | 0 | 3 | 2 | m | - | no |
| *Cryptophagus scanicus* | (Linnaeus, 1758) | Cryptophagidae | 4 | 0 | 2 | m | - | no |
| *Cryptophagus scutellatus* | Newman, 1834 | Cryptophagidae | 0 | 2 | 2 | m | - | no |
| *Cryptophagus subdepressus* | Gyllenhal, 1827 | Cryptophagidae | 1 | 0 | 1 | m | - | no |
| *Cryptophagus uncinatus* | Stephens, 1830 | Cryptophagidae | 0 | 1 | 3 | m | - | no |
| *Ephistemus reitteri* | Ephistemus reitteri | Cryptophagidae | 0 | 8 | 3 | m | - | no |
| *Micrambe abietis* | Micrambe abietis | Cryptophagidae | 7 | 0 | 1 | m | - | no |
| *Micrambe bimaculata* | Micrambe bimaculata | Cryptophagidae | 0 | 1 | 3 | m | - | no |
| *Paramecosoma melanocephalum* | Paramecosoma melanocephalum | Cryptophagidae | 0 | 4 | 2 | m | - | no |
| *Pteryngium crenatum* | Pteryngium crenatum | Cryptophagidae | 5 | 0 | 1 | m | - | no |
| *Cucujus cinnaberinus* | Cucujus cinnaberinus | Cucujidae | 0 | 4 | 1 | z | EN | yes |
| *Pediacus dermestoides* | Pediacus dermestoides | Cucujidae | 3 | 0 | 1 | z | VU | yes |
| *Acalles camelus* | (Fabricius, 1792) | Curculionidae | 1 | 0 | 1 | x | NT | no |
| *Acalles fallax* | Boheman, 1844 | Curculionidae | 1 | 0 | 1 | x | NT | no |
| *Camptorhinus statua* | (Rossi, 1790) | Curculionidae | 0 | 4 | 1 | x | EN | yes |
| *Cossonus parallelepipedus* | (Herbst, 1795) | Curculionidae | 0 | 6 | 1 | x | - | no |
| *Gasterocercus depressirostris* | (Fabricius, 1792) | Curculionidae | 0 | 11 | 1 | x | VU | yes |
| *Hexarthrum exiguum* | (Boheman, 1838) | Curculionidae | 0 | 8 | 1 | x | - | no |
| *Magdalis armigera* | (Geoffroy, 1785) | Curculionidae | 0 | 5 | 1 | x | - | no |
| *Magdalis barbicornis* | (Latreille, 1804) | Curculionidae | 0 | 3 | 2 | x | - | no |
| *Melicius cylindrus* | (Boheman, 1838) | Curculionidae | 0 | 4 | 1 | x | NT | no |
| *Pissodes piceae* | (Illiger, 1807) | Curculionidae | 1 | 0 | 1 | x | - | no |
| *Rhyncolus ater* | (Linnaeus, 1758) | Curculionidae | 2 | 9 | 1 | x | - | no |
| *Rhyncolus reflexus* | Boheman, 1838 | Curculionidae | 0 | 1 | 1 | x | VU | no |
| *Rhyncolus sculpturatus* | Waltl, 1839 | Curculionidae | 0 | 2 | 1 | x | NT | no |
| *Stereocorynes truncorum* | (Germar, 1824) | Curculionidae | 0 | 2 | 1 | x | - | no |
| *Aplocnemus impressus* | (Marsham, 1802) | Dasytidae | 0 | 1 | 1 | z | - | no |
| *Dasytes aeratus* | Stephens, 1829 | Dasytidae | 0 | 67 | 1 | z | - | no |
| *Dasytes caeruleus* | (DeGeer, 1774) | Dasytidae | 0 | 4 | 1 | z | - | no |
| *Dasytes plumbeus* | (O. F. Müller 1776) | Dasytidae | 0 | 22 | 1 | z | - | no |
| *Anthrenus pimpinellae* | (Fabricius, 1775) | Dermestidae | 0 | 2 | 2 | s | - | no |
| *Anthrenus scrophulariae* | (Linnaeus, 1758) | Dermestidae | 0 | 1 | 2 | s | - | no |
| *Attagenus punctatus* | (Scopoli, 1772) | Dermestidae | 0 | 3 | 3 | s | - | yes |
| *Attagenus schaefferi* | (Herbst, 1792) | Dermestidae | 0 | 3 | 3 | s | - | no |
| *Ctesias serra* | (Fabricius, 1792) | Dermestidae | 1 | 2 | 3 | s | - | no |
| *Globicornis nigripes* | (Fabricius, 1792) | Dermestidae | 0 | 14 | 2 | s | - | no |
| *Megatoma undata* | (Linnaeus, 1758) | Dermestidae | 0 | 30 | 3 | s | - | no |
| *Orphilus niger* | (P. Rossi, 1790) | Dermestidae | 0 | 4 | 2 | s | - | no |
| *Trinodes hirtus* | (Fabricius, 1781) | Dermestidae | 0 | 7 | 3 | s | - | no |
| *Dryophthorus corticalis* | (Paykull, 1792) | Dryophtoridae | 0 | 45 | 1 | x | - | no |
| *Ampedus aethiops* | (Lacordaire, 1835) | Elateridae | 2 | 0 | 1 | z | - | no |
| *Ampedus cardinalis* | (Schiödte, 1865) | Elateridae | 0 | 10 | 1 | z | VU | yes |
| *Ampedus elongatulus* | Fabricius, 1787 | Elateridae | 0 | 13 | 1 | z | - | yes |
| *Ampedus erythrogonus* | (P. W. Müller, 1821) | Elateridae | 59 | 0 | 1 | z | - | no |
| *Ampedus melanurus* | Mulsant & Guillebeau, 1855 | Elateridae | 2 | 0 | 1 | z | CR | yes |
| *Ampedus nigerrimus* | (Lacordaire, 1835) | Elateridae | 0 | 19 | 1 | z | EN | yes |
| *Ampedus nigrinus* | (Herbst, 1784) | Elateridae | 33 | 0 | 1 | z | - | no |
| *Ampedus pomonae* | (Stephens, 1830) | Elateridae | 0 | 2 | 1 | z | NT | no |
| *Ampedus pomorum* | (Herbst, 1784) | Elateridae | 6 | 167 | 1 | z | - | no |
| *Ampedus praeustus* | (Fabricius, 1792) | Elateridae | 0 | 4 | 1 | z | VU | yes |
| *Ampedus rufipennis* | (Stephens, 1830) | Elateridae | 0 | 38 | 1 | z | VU | no |
| *Ampedus sanguineus* | (Linnaeus, 1758) | Elateridae | 1 | 0 | 1 | z | - | no |
| *Ampedus sanguinolentus* | (Schrank, 1776) | Elateridae | 0 | 132 | 1 | z | NT | no |
| *Ampedus sinuatus* | Germar, 1844 | Elateridae | 0 | 4 | 1 | z | VU | yes |
| *Brachygonus bouyoni* | Chassain, 1992 | Elateridae | 0 | 3 | 1 | z | CR | no |
| *Brachygonus megerlei* | (Lacordaire, 1835) | Elateridae | 0 | 1 | 1 | z | VU | yes |
| *Brachygonus ruficeps* | (Mulsant & Guillebeau, 1855) | Elateridae | 0 | 6 | 1 | z | CR | yes |
| *Calambus bipustulatus* | (Linnaeus, 1767) | Elateridae | 0 | 9 | 1 | z | NT | no |
| *Cardiophorus gramineus* | (Scopoli, 1763) | Elateridae | 0 | 3 | 2 | z | VU | yes |
| *Crepidophorus mutilatus* | (Rosenhauer, 1847) | Elateridae | 3 | 0 | 1 | z | CR | yes |
| *Denticollis interpositus* | Roubal, 1941 | Elateridae | 3 | 0 | 1 | z | EN | no |
| *Denticollis linearis* | (Linnaeus, 1758) | Elateridae | 15 | 28 | 1 | z | - | no |
| *Denticollis rubens* | Piller & Mitterpacher, 1783 | Elateridae | 22 | 0 | 1 | z | VU | yes |
| *Diacanthous undulatus* | (DeGeer, 1774) | Elateridae | 2 | 0 | 1 | z | EN | yes |
| *Elater ferrugineus* | Linnaeus, 1758 | Elateridae | 0 | 2 | 1 | z | CR | yes |
| *Ischnodes sanguinicollis* | (Panzer, 1793) | Elateridae | 0 | 1 | 1 | z | CR | yes |
| *Megapenthes lugens* | (L. Redtenbacher, 1842) | Elateridae | 0 | 2 | 1 | z | CR | yes |
| *Melanotus castanipes* | (Paykull, 1800) | Elateridae | 60 | 21 | 1 | z | - | no |
| *Podeonius acuticornis* | (Germar, 1824) | Elateridae | 0 | 2 | 1 | z | CR | yes |
| *Procraerus tibialis* | (Lacordaire, 1835) | Elateridae | 2 | 1 | 1 | z | - | yes |
| *Reitterelater dubius* | Platia & Cate, 1990 | Elateridae | 0 | 20 | 1 | z | CR | yes |
| *Stenagostus rhombeus* | (A. G. Olivier, 1790) | Elateridae | 0 | 37 | 1 | z | EN | yes |
| *Clemmus troglodytes* | Hampe, 1850 | Endomychidae | 0 | 4 | 3 | m | CR | no |
| *Endomychus coccineus* | (Linnaeus, 1758) | Endomychidae | 6 | 1 | 3 | m | VU | no |
| *Holoparamecus caularum* | Aubé, 1843 | Endomychidae | 0 | 1 | 3 | m | - | no |
| *Mycetina cruciata* | (Schaller, 1783) | Endomychidae | 32 | 7 | 2 | m | - | yes |
| *Symbiotes gibberosus* | (P. H. Lucas, 1846) | Endomychidae | 0 | 91 | 2 | m | - | yes |
| *Symbiotes latus* | L. Redtenbacher, 1849 | Endomychidae | 0 | 24 | 2 | m | CR | yes |
| *Dacne bipustulata* | (Thunberg, 1781) | Erotylidae | 0 | 26 | 2 | m | - | no |
| *Triplax aenea* | (Schaller, 1783) | Erotylidae | 16 | 1 | 2 | m | - | yes |
| *Triplax collaris* | (Schaller, 1783) | Erotylidae | 0 | 3 | 2 | m | VU | yes |
| *Triplax elongata* | Lacordaire, 1842 | Erotylidae | 0 | 2 | 2 | m | EN | yes |
| *Triplax russica* | (Linnaeus, 1758) | Erotylidae | 55 | 0 | 2 | m | - | no |
| *Tritoma bipustulata* | Fabricius, 1775 | Erotylidae | 0 | 44 | 2 | m | - | no |
| *Eucinetus hopffgarteni* | (Reitter, 1885) | Eucinetidae | 1 | 0 | 2 | m | - | no |
| *Dirrhagofarsus attenuatus* | (Mäklin, 1845) | Eucnemidae | 0 | 2 | 1 | m | CR | no |
| *Dromaeolus barnabita* | (A. Villa & J.B. Villa, 1838) | Eucnemidae | 0 | 14 | 1 | m | EN | yes |
| *Eucnemis capucina* | Ahrens, 1812 | Eucnemidae | 1 | 2 | 1 | m | - | no |
| *Hylis cariniceps* | (Reitter, 1902) | Eucnemidae | 0 | 2 | 1 | m | CR | no |
| *Hylis foveicollis* | (C.G. Thomson, 1874) | Eucnemidae | 4 | 0 | 1 | m | EN | no |
| *Hylis olexai* | (Palm, 1955) | Eucnemidae | 0 | 5 | 1 | m | CR | no |
| *Hylis procerulus* | (Mannerheim, 1823) | Eucnemidae | 0 | 9 | 1 | m | CR | yes |
| *Hylis simonae* | (Olexa, 1970) | Eucnemidae | 0 | 3 | 1 | m | - | no |
| *Isorhipis marmottani* | (Bonvouloir, 1871) | Eucnemidae | 0 | 38 | 1 | m | CR | yes |
| *Isorhipis melasoides* | (Laporte de Castelnau, 1835) | Eucnemidae | 0 | 1 | 1 | m | EN | no |
| *Melasis buprestoides* | (Linnaeus, 1761) | Eucnemidae | 9 | 45 | 1 | m | - | no |
| *Microrhagus emyi* | (Rouget, 1856) | Eucnemidae | 0 | 4 | 1 | m | CR | yes |
| *Microrhagus lepidus* | Rosenhauer, 1847 | Eucnemidae | 1 | 5 | 1 | m | EN | no |
| *Microrhagus pygmaeus* | (Fabricius, 1792) | Eucnemidae | 0 | 1 | 1 | m | EN | no |
| *Xylophilus corticalis* | (Paykull, 1800) | Eucnemidae | 1 | 0 | 1 | m | CR | yes |
| *Xylophilus testaceus* | (Herbst, 1806) | Eucnemidae | 0 | 3 | 1 | m | CR | yes |
| *Abraeus granulum* | Erichson, 1839 | Histeridae | 3 | 0 | 1 | z | VU | no |
| *Abraeus perpusillus* | (Marsham, 1802) | Histeridae | 0 | 15 | 1 | z | - | no |
| *Acritus minutus* | (Herbst, 1792) | Histeridae | 0 | 5 | 1 | z | - | no |
| *Acritus nigricornis* | (Hoffman, 1803) | Histeridae | 0 | 1 | 1 | z | - | no |
| *Carcinops pumilio* | (Erichson, 1834) | Histeridae | 0 | 1 | 3 | z | - | no |
| *Gnathoncus nannetensis* | (Marseul, 1862) | Histeridae | 2 | 1 | 2 | z | - | no |
| *Hololepta plana* | (Sulzer, 1776) | Histeridae | 0 | 1 | 1 | z | - | no |
| *Paromalus flavicornis* | (Herbst, 1792) | Histeridae | 0 | 13 | 1 | z | - | no |
| *Paromalus parallelepipedus* | (Herbst, 1792) | Histeridae | 0 | 1 | 1 | z | - | no |
| *Plegaderus caesus* | (Herbst, 1792) | Histeridae | 1 | 0 | 1 | z | - | no |
| *Cryptolestes duplicatus* | (Waltl, 1834) | Laemophloeidae | 0 | 8 | 2 | s | - | no |
| *Cryptolestes ferrugineus* | (Stephens, 1831) | Laemophloeidae | 0 | 1 | 2 | s | - | no |
| *Laemophloeus monilis* | (Fabricius, 1787) | Laemophloeidae | 0 | 1 | 1 | z | - | no |
| *Lathropus sepicola* | (P. W. J. Müller, 1821) | Laemophloeidae | 0 | 7 | 2 | s | VU | no |
| *Leptophloeus alternans* | (Erichson, 1846) | Laemophloeidae | 3 | 1 | 1 | z | - | no |
| *Placonotus testaceus* | (Fabricius, 1787) | Laemophloeidae | 0 | 5 | 2 | s | - | no |
| *Cartodere (Aridius) nodifer* | (Westwood, 1839) | Latrididae | 1 | 49 | 3 | m | - | no |
| *Corticaria bella* | L. Redtenbacher, 1849 | Latrididae | 0 | 67 | 2 | m | - | no |
| *Corticaria elongata* | (Gyllenhal, 1827) | Latrididae | 0 | 2 | 3 | m | - | no |
| *Corticaria lapponica* | (Zetterstedt, 1838) | Latrididae | 0 | 5 | 2 | m | - | no |
| *Corticaria longicornis* | (Herbst, 1783) | Latrididae | 3 | 0 | 2 | m | - | no |
| *Corticaria rubripes* | Mannerheim, 1844 | Latrididae | 1 | 0 | 2 | m | - | no |
| *Corticaria serrata* | (Paykull, 1798) | Latrididae | 0 | 5 | 2 | m | - | no |
| *Corticarina minuta* | (Fabricius, 1792) | Latrididae | 9 | 22 | 3 | m | - | no |
| *Corticarina parvula* | (Mannerheim, 1844) | Latridiidae | 26 | 0 | 3 | m | - | no |
| *Corticarina similata* | (Gyllenhal, 1827) | Latridiidae | 24 | 7 | 3 | m | - | no |
| *Dienerella costulata* | (Reitter, 1877) | Latridiidae | 0 | 6 | 1 | m | - | no |
| *Enicmus atriceps* | V. Hansen, 1962 | Latridiidae | 3 | 360 | 2 | m | - | no |
| *Enicmus brevicornis* | (Mannerheim, 1844) | Latridiidae | 0 | 1 | 2 | m | - | no |
| *Enicmus fungicola* | C. G. Thomson, 1868 | Latridiidae | 18 | 8 | 2 | m | - | no |
| *Enicmus histrio* | Joy & Tomlin, 1910 | Latridiidae | 1 | 13 | 3 | m | - | no |
| *Enicmus rugosus* | (Herbst, 1793) | Latridiidae | 13 | 545 | 2 | m | - | no |
| *Enicmus testaceus* | (Stephens, 1830) | Latridiidae | 39 | 0 | 2 | m | - | no |
| *Enicmus transversus* | (A. G. Olivier, 1790) | Latridiidae | 1 | 14 | 3 | m | - | no |
| *Latridius brevicollis* | (C. G. Thomson, 1868) | Latridiidae | 3 | 0 | 1 | m | - | no |
| *Latridius consimilis* | (Mannerheim, 1844) | Latridiidae | 7 | 0 | 2 | m | - | no |
| *Latridius hirtus* | (Gyllenhal, 1827) | Latridiidae | 5 | 122 | 2 | m | - | no |
| *Latridius minutus* | (Linnaeus, 1767) | Latridiidae | 4 | 1 | 2 | m | - | no |
| *Latridius porcatus* | Herbst, 1793 | Latridiidae | 3 | 0 | 2 | m | - | no |
| *Stephostethus alternans* | (Mannerheim, 1844) | Latridiidae | 12 | 5 | 2 | m | - | no |
| *Stephostethus angusticollis* | (Gyllenhal, 1827) | Latridiidae | 1 | 5 | 2 | m | - | no |
| *Stephostethus caucasicus* | (Mannerheim, 1844) | Latridiidae | 0 | 1 | 2 | m | - | no |
| *Stephostethus rugicollis* | (A. G. Olivier, 1790) | Latridiidae | 15 | 0 | 2 | m | - | no |
| *Agathidium nigrinum* | Sturm, 1807 | Leiodidae | 1 | 0 | 2 | m | - | no |
| *Agathidium nigripenne* | (Fabricius, 1792) | Leiodidae | 9 | 6 | 1 | m | - | no |
| *Agathidium plagiatum* | (Gyllenhal, 1810) | Leiodidae | 18 | 0 | 2 | m | - | no |
| *Agathidium varians* | Beck, 1817 | Leiodidae | 3 | 0 | 2 | m | - | no |
| *Amphicyllis globiformis* | (Sahlberg, 1833) | Leiodidae | 2 | 0 | 2 | m | - | no |
| *Anisotoma castanea* | (Herbst, 1792) | Leiodidae | 11 | 0 | 1 | m | - | no |
| *Anisotoma humeralis* | (Fabricius, 1792) | Leiodidae | 86 | 101 | 1 | m | - | no |
| *Anisotoma orbicularis* | (Herbst, 1792) | Leiodidae | 4 | 29 | 1 | m | - | no |
| *Liodopria serricornis* | (Gyllenhal, 1813) | Leiodidae | 1 | 0 | 2 | m | VU | no |
| *Nemadus colonoides* | (Kraatz, 1851) | Leiodidae | 0 | 5 | 3 | s | NT | no |
| *Aesalus scarabaeoides* | (Panzer, 1794) | Lucanidae | 0 | 15 | 1 | x | - | yes |
| *Ceruchus chrysomelinus* | (Hochenwarth, 1785) | Lucanidae | 1 | 0 | 1 | x | CR | yes |
| *Dorcus parallelipipedus* | (Linnaeus, 1758) | Lucanidae | 0 | 2 | 1 | x | - | no |
| *Lucanus cervus* | (Linnaeus, 1758) | Lucanidae | 0 | 2 | 1 | x | EN | no |
| *Platycerus caraboides* | (Linnaeus, 1758) | Lucanidae | 8 | 0 | 1 | x | - | no |
| *Sinodendron cylindricum* | (Linnaeus, 1758) | Lucanidae | 1 | 4 | 1 | x | - | no |
| *Dictyoptera aurora* | (Herbst, 1784) | Lycidae | 6 | 0 | 1 | z | - | no |
| *Platycis minutus* | (Fabricius, 1787) | Lycidae | 0 | 1 | 1 | z | - | no |
| *Pyropterus nigroruber* | (DeGeer, 1774) | Lycidae | 8 | 0 | 1 | z | - | no |
| *Elateroides dermestoides* | (Linnaeus, 1761) | Lymexylonidae | 91 | 0 | 1 | m | - | no |
| *Lymexylon navale* | (Linnaeus, 1758) | Lymexylonidae | 1 | 5 | 1 | m | VU | yes |
| *Anthocomus humeralis* | (Morawitz, 1862) | Malachiidae | 0 | 2 | 3 | z | - | no |
| *Axinotarsus ruficollis* | (A. G. Olivier, 1790) | Malachiidae | 0 | 5 | 1 | z | VU | no |
| *Malachius bipustulatus* | (Linnaeus, 1758) | Malachiidae | 0 | 6 | 1 | z | - | no |
| *Abdera flexuosa* | (Paykull, 1799) | Melandryidae | 8 | 0 | 1 | m | - | no |
| *Anisoxya fuscula* | (Illiger, 1798) | Melandryidae | 0 | 1 | 1 | m | VU | no |
| *Conopalpus testaceus* | (A.G. Olivier, 1790) | Melandryidae | 3 | 2 | 1 | m | VU | no |
| *Dircaea australis* | Fairmaire, 1856 | Melandryidae | 0 | 20 | 1 | m | CR | yes |
| *Dolotarsus lividus* | (C. R. Sahlberg, 1833) | Melandryidae | 4 | 0 | 1 | m | - | yes |
| *Eustrophus dermestoides* | (Fabricius, 1792) | Melandryidae | 0 | 1 | 1 | m | - | yes |
| *Hypulus quercinus* | (Quensel, 1790) | Melandryidae | 0 | 3 | 1 | m | EN | yes |
| *Melandrya barbata* | (Fabricius, 1787) | Melandryidae | 1 | 2 | 1 | m | VU | no |
| *Melandrya caraboides* | (Linnaeus, 1760) | Melandryidae | 0 | 1 | 1 | m | EN | no |
| *Melandrya dubia* | (Schaller, 1783) | Melandryidae | 2 | 0 | 1 | m | - | no |
| *Orchesia micans* | (Panzer, 1793) | Melandryidae | 33 | 2 | 1 | m | - | no |
| *Orchesia minor* | Walker, 1837 | Melandryidae | 4 | 0 | 1 | m | - | no |
| *Orchesia undulata* | Kraatz, 1853 | Melandryidae | 7 | 2 | 1 | m | VU | no |
| *Osphya bipunctata* | (Fabricius, 1775) | Melandryidae | 0 | 4 | 1 | m | - | yes |
| *Phloiotrya rufipes* | (Gyllenhal, 1810) | Melandryidae | 2 | 0 | 1 | m | - | no |
| *Rhizophagus bipustulatus* | (Fabricius, 1792) | Monotomidae | 13 | 42 | 2 | s | - | no |
| *Rhizophagus cribratus* | Gyllenhal, 1827 | Monotomidae | 0 | 1 | 2 | s | VU | no |
| *Rhizophagus dispar* | (Paykull, 1800) | Monotomidae | 4 | 0 | 2 | s | - | no |
| *Rhizophagus fenestralis* | (Linnaeus, 1758) | Monotomidae | 0 | 3 | 2 | s | - | no |
| *Rhizophagus ferrugineus* | (Paykull, 1800) | Monotomidae | 1 | 0 | 2 | s | - | no |
| *Rhizophagus nitidulus* | (Fabricius, 1798) | Monotomidae | 5 | 0 | 2 | s | - | no |
| *Rhizophagus perforatus* | Erichson, 1845 | Monotomidae | 1 | 8 | 2 | s | - | no |
| *Rhizophagus picipes* | (A. G. Olivier, 1790) | Monotomidae | 0 | 1 | 2 | s | - | no |
| *Hoshihananomia gacognei* | (Mulsant, 1852) | Mordellidae | 0 | 2 | 1 | m | CR | no |
| *Mordellaria aurofasciata* | (Comolli, 1837) | Mordellidae | 0 | 5 | 1 | m | - | no |
| *Mordellistena humeralis* | (Fabricius, 1758) | Mordellidae | 0 | 3 | 1 | m | CR | no |
| *Mordellistena neuwaldeggiana* | (Panzer, 1796) | Mordellidae | 0 | 5 | 1 | m | - | no |
| *Mordellistena variegata* | (Fabricius, 1798) | Mordellidae | 0 | 6 | 1 | m | - | no |
| *Mordellochroa abdominalis* | Fabricius, 1775 | Mordellidae | 0 | 10 | 1 | m | - | no |
| *Mordellochroa milleri* | (Emery, 1876) | Mordellidae | 0 | 3 | 1 | m | CR | no |
| *Tomoxia bucephala* | (A. Costa, 1854) | Mordellidae | 40 | 57 | 1 | m | - | no |
| *Variimorda villosa* | (Schrank von Paula, 1781) | Mordellidae | 0 | 1 | 1 | m | - | no |
| *Litargus connexus* | (Geoffroy, 1785) | Mycetophagidae | 1 | 77 | 2 | m | - | no |
| *Mycetophagus atomarius* | (Fabricius, 1787) | Mycetophagidae | 3 | 3 | 1 | m | - | no |
| *Mycetophagus fulvicollis* | Fabricius, 1793 | Mycetophagidae | 0 | 48 | 1 | m | VU | yes |
| *Mycetophagus piceus* | (Fabricius, 1777) | Mycetophagidae | 0 | 2 | 1 | m | - | no |
| *Mycetophagus populi* | Fabricius, 1798 | Mycetophagidae | 0 | 1 | 1 | m | VU | yes |
| *Mycetophagus quadripustulatus* | (Linnaeus, 1761) | Mycetophagidae | 1 | 5 | 2 | m | - | no |
| *Triphyllus bicolor* | (Fabricius, 1777) | Mycetophagidae | 7 | 0 | 1 | m | VU | no |
| *Cryptarcha strigata* | (Fabricius, 1787) | Nitidulidae | 0 | 1 | 2 | m | - | no |
| *Cychramus luteus* | (Fabricius, 1787) | Nitidulidae | 18 | 0 | 2 | m | - | no |
| *Cychramus variegatus* | (Herbst, 1792) | Nitidulidae | 47 | 0 | 1 | m | - | no |
| *Cyllodes ater* | (Herbst, 1792) | Nitidulidae | 14 | 0 | 1 | m | VU | no |
| *Epuraea angustula* | Sturm, 1844 | Nitidulidae | 2 | 0 | 1 | m | - | no |
| *Epuraea biguttata* | (Thunberg, 1784) | Nitidulidae | 0 | 1 | 2 | m | - | no |
| *Epuraea laeviuscula* | (Gyllenhal, 1827) | Nitidulidae | 2 | 0 | 1 | m | VU | no |
| *Epuraea marseuli* | Reitter, 1873 | Nitidulidae | 3 | 0 | 1 | m | - | no |
| *Epuraea melanocephala* | (Marsham, 1802) | Nitidulidae | 47 | 102 | 2 | m | - | no |
| *Epuraea neglecta* | (Heer, 1841) | Nitidulidae | 6 | 1 | 2 | m | - | no |
| *Epuraea pallescens* | (Stephens, 1835) | Nitidulidae | 1 | 1 | 2 | m | - | no |
| *Epuraea silacea* | (Herbst, 1783) | Nitidulidae | 1 | 0 | 1 | m | - | no |
| *Epuraea unicolor* | (A. G. Olivier, 1790) | Nitidulidae | 6 | 0 | 3 | m | - | no |
| *Epuraea variegata* | (Herbst, 1793) | Nitidulidae | 3 | 1 | 2 | m | - | no |
| *Glischrochilus quadriguttatus* | (Fabricius, 1777) | Nitidulidae | 0 | 12 | 1 | m | - | no |
| *Glischrochilus quadripunctatus* | (Linnaeus, 1758) | Nitidulidae | 1 | 1 | 3 | m | - | no |
| *Pityophagus ferrugineus* | (Linnaeus, 1761) | Nitidulidae | 2 | 0 | 1 | z | - | no |
| *Phloeostichus denticollis* | W. Redtenbacher, 1842 | Phloeostichidae | 1 | 0 | 2 | z | EN | yes |
| *Platypus cylindrus* | (Fabricius, 1792) | Platypodinae | 0 | 3 | 1 | m | - | no |
| *Prostomis mandibularis* | (Fabricius, 1801) | Prostomidae | 0 | 34 | 1 | x | CR | yes |
| *Dorcatoma chrysomelina* | Sturm, 1837 | Ptinidae | 0 | 18 | 1 | m | - | yes |
| *Dorcatoma dresdensis* | Herbst, 1792 | Ptinidae | 6 | 5 | 1 | m | - | no |
| *Dorcatoma robusta* | A. Strand, 1938 | Ptinidae | 1 | 1 | 1 | m | - | yes |
| *Dorcatoma substriata serra* | Panzer, 1795 | Ptinidae | 1 | 0 | 1 | m | - | no |
| *Dryophillus pusillus* | (Gyllenhal, 1808) | Ptinidae | 3 | 0 | 1 | x | - | no |
| *Episernus striatellus* | (C. N. F. Brisout de Barneville, 1863) | Ptinidae | 5 | 0 | 1 | x | - | no |
| *Ernobius abietis* | (Fabricius, 1792) | Ptinidae | 1 | 0 | 1 | x | - | no |
| *Ernobius mollis mollis* | (Linnaeus, 1758) | Ptinidae | 6 | 0 | 1 | x | - | no |
| *Gastrallus immarginatus* | (P. W. J. Müller, 1821) | Ptinidae | 0 | 1 | 1 | x | - | no |
| *Gastrallus knizeki* | Zahradník, 1996 | Ptinidae | 0 | 1 | 2 | x | - | yes |
| *Gastrallus laevigatus* | (A. G. Olivier, 1790) | Ptinidae | 0 | 43 | 2 | x | - | yes |
| *Hadrobregmus pertinax* | (Linnaeus, 1758) | Ptinidae | 5 | 0 | 1 | x | - | no |
| *Hedobia pubescens* | (A. G. Olivier, 1790) | Ptinidae | 0 | 3 | 1 | x | - | yes |
| *Hemicoelus canaliculatus* | C. G. Thomson, 1863 | Ptinidae | 2 | 4 | 1 | x | - | no |
| *Hemicoelus costatus* | (Aragona, 1830) | Ptinidae | 48 | 1 | 1 | x | - | no |
| *Hemicoelus fulvicornis* | (Sturm, 1837) | Ptinidae | 2 | 12 | 1 | x | - | no |
| *Hemicoelus rufipennis* | (Duftschmid, 1825) | Ptinidae | 0 | 9 | 1 | x | - | no |
| *Hyperisus plumbeum* | (Illiger, 1801) | Ptinidae | 37 | 0 | 1 | x | - | no |
| *Microbregma emarginatum* | (Duftschmid, 1825) | Ptinidae | 3 | 0 | 1 | x | - | no |
| *Oligomerus brunneus* | (A. G. Olivier, 1790) | Ptinidae | 0 | 11 | 1 | x | - | no |
| *Oligomerus retowskii* | Schilsky, 1898 | Ptinidae | 0 | 17 | 1 | x | - | no |
| *Priobium carpini* | (Herbst, 1793) | Ptinidae | 0 | 8 | 1 | x | - | no |
| *Pseudoptilinus fissicollis* | (Reitter, 1877) | Ptinidae | 0 | 53 | 1 | x | - | no |
| *Ptilinus fuscus* | (Geoffroy, 1785) | Ptinidae | 0 | 5 | 1 | x | - | no |
| *Ptilinus pectinicornis* | (Linnaeus, 1758) | Ptinidae | 99 | 4 | 1 | x | - | no |
| *Ptinomorphus imperialis* | (Linnaeus, 1767) | Ptinidae | 31 | 3 | 1 | x | - | no |
| *Ptinomorphus regalis* | (Duftschmid, 1825) | Ptinidae | 0 | 12 | 1 | x | - | no |
| *Ptinus rufipes* | A. G. Olivier, 1790 | Ptinidae | 0 | 4 | 1 | s | - | no |
| *Ptinus schlerethi* | (Reitter, 1884) | Ptinidae | 0 | 1 | 2 | s | EN | no |
| *Ptinus sexpunctatus* | Panzer, 1789 | Ptinidae | 0 | 3 | 2 | s | - | no |
| *Ptinus subpillosus* | Sturm, 1837 | Ptinidae | 3 | 2 | 2 | s | - | no |
| *Stagetus pilula* | (Aubé, 1861) | Ptinidae | 0 | 8 | 1 | m | - | no |
| *Xyletinus ater* | (Creutzer, 1796) | Ptinidae | 0 | 2 | 1 | x | - | no |
| *Xyletinus longitarsis longitarsis* | Jansson, 1942 | Ptinidae | 0 | 7 | 1 | x | - | no |
| *Pyrochroa coccinea* | (Linnaeus, 1761) | Pyrochroidae | 1 | 4 | 1 | z | - | no |
| *Pyrochroa serraticornis* | (Scopoli, 1763) | Pyrochroidae | 0 | 8 | 1 | z | - | no |
| *Schizotus pectinicornis* | (Linnaeus, 1758) | Pyrochroidae | 2 | 19 | 1 | z | - | no |
| *Pelecotoma fennica* | (Paykull, 1799) | Ripiphoridae | 0 | 1 | 1 | z | CR | no |
| *Lissodema cursor* | (Gyllenhal, 1813) | Salpingidae | 0 | 7 | 1 | s | - | no |
| *Lissodema denticolle* | (Gyllenhal, 1813) | Salpingidae | 0 | 12 | 1 | s | - | no |
| *Rabocerus foveolatus* | (Ljungh, 1823) | Salpingidae | 102 | 0 | 1 | s | VU | no |
| *Salpingus planirostris* | (Fabricius, 1787) | Salpingidae | 13 | 12 | 1 | s | - | no |
| *Salpingus ruficollis* | (Linnaeus, 1761) | Salpingidae | 42 | 13 | 1 | s | - | no |
| *Vincenzellus ruficollis* | (Panzer, 1794) | Salpingidae | 0 | 20 | 1 | s | - | no |
| *Cetonia aurata* | (Linnaeus, 1761) | Scarabaeidae | 0 | 1 | 2 | s | - | no |
| *Gnorimus variabilis* | (Linnaeus, 1758) | Scarabaeidae | 0 | 1 | 1 | x | EN | yes |
| *Protaetia aeruginosa* | (Drury, 1770) | Scarabaeidae | 0 | 1 | 1 | x | EN | yes |
| *Protaetia cuprea* | (Fabricius, 1775) | Scarabaeidae | 0 | 3 | 2 | s | - | yes |
| *Protaetia lugubris* | (Herbst, 1786) | Scarabaeidae | 0 | 2 | 1 | s | - | no |
| *Valgus hemipterus* | (Linnaeus, 1758) | Scarabaeidae | 0 | 3 | 1 | x | - | no |
| *Prionocyphon serricornis* | (P. W. J. Müller, 1821) | Scirtidae | 3 | 0 | 3 | s | VU | no |
| *Cryphalus abietis* | (Ratzeburg, 1837) | Scolytinae | 933 | 0 | 1 | x | - | no |
| *Cryphalus piceae* | (Ratzeburg, 1837) | Scolytinae | 3 | 0 | 1 | x | - | no |
| *Crypturgus hispidulus* | Thomson, 1870 | Scolytinae | 16 | 0 | 1 | x | - | no |
| *Crypturgus pusillus* | (Gyllenhal, 1813) | Scolytinae | 0 | 1 | 1 | x | - | no |
| *Crypturgus subcribrosus* | Eggers, 1933 | Scolytinae | 24 | 0 | 1 | x | - | no |
| *Dryocoetes alni* | (Georg, 1856) | Scolytinae | 1 | 0 | 1 | x | - | no |
| *Dryocoetes autographus* | (Ratzeburg, 1837) | Scolytinae | 49 | 0 | 1 | x | - | no |
| *Dryocoetes hectographus* | Reitter, 1913 | Scolytinae | 18 | 0 | 1 | x | - | no |
| *Dryocoetes villosus* | (Fabricius, 1792) | Scolytinae | 0 | 6 | 1 | x | - | no |
| *Ernoporicus fagi* | (Fabricius, 1798) | Scolytinae | 3188 | 0 | 1 | x | - | no |
| *Ernoporus tiliae* | (Panzer, 1793) | Scolytinae | 2 | 3 | 1 | x | - | no |
| *Hylastes cunicularius* | Erichson, 1836 | Scolytinae | 17 | 0 | 1 | x | - | no |
| *Hylastes opacus* | Erichson, 1836 | Scolytinae | 0 | 1 | 1 | x | - | no |
| *Hylesinus crenatus* | (Fabricius, 1787) | Scolytinae | 0 | 43 | 1 | x | - | no |
| *Hylesinus fraxini* | (Panzer, 1779) | Scolytinae | 2 | 545 | 1 | x | - | no |
| *Hylesinus toranio* | (Danthoine,1788) | Scolytinae | 1 | 203 | 1 | x | - | no |
| *Hylesinus wachtli orni* | Reitter, 1887 | Scolytinae | 0 | 32 | 1 | x | - | no |
| *Hylurgops palliatus* | (Gyllenhal, 1813) | Scolytinae | 55 | 0 | 1 | x | - | no |
| *Ips typographus* | (Linnaeus, 1758) | Scolytinae | 22 | 0 | 1 | x | - | no |
| *Lymantor coryli* | (Perris, 1853) | Scolytinae | 0 | 1 | 1 | x | - | no |
| *Phloeotribus caucasicus* | Reitter, 1891 | Scolytinae | 0 | 2 | 1 | x | - | no |
| *Phloeotribus spinulosus* | (Rey, 1883) | Scolytinae | 7 | 0 | 1 | x | - | no |
| *Pityogenes chalcographus* | (Linnaeus, 1761) | Scolytinae | 60 | 1 | 1 | x | - | no |
| *Pityophthorus pityographus* | (Ratzeburg, 1837) | Scolytinae | 31 | 0 | 1 | x | - | no |
| *Polygraphus poligraphus* | (Linnaeus, 1758) | Scolytinae | 5 | 0 | 1 | x | - | no |
| *Pteleobius vittatus* | (Fabricius, 1787) | Scolytinae | 0 | 4 | 1 | x | VU | no |
| *Scolytus carpini* | (Ratzeburg, 1837) | Scolytinae | 0 | 47 | 1 | x | - | no |
| *Scolytus ensifer* | Eichhof, 1881 | Scolytinae | 0 | 17 | 1 | x | VU | no |
| *Scolytus intricatus* | (Ratzeburg, 1837) | Scolytinae | 0 | 6 | 1 | x | - | no |
| *Scolytus kirschii* | Skalitzky, 1876 | Scolytinae | 0 | 2 | 1 | x | VU | no |
| *Scolytus multistriatus* | (Marsham, 1802) | Scolytinae | 2 | 116 | 1 | x | VU | no |
| *Scolytus pygmaeus* | (Fabricius, 1787) | Scolytinae | 0 | 1 | 1 | x | VU | no |
| *Scolytus ratzeburgii* | Janson, 1856 | Scolytinae | 0 | 2 | 1 | x | - | no |
| *Scolytus rugulosus* | (P. W. J. Müller, 1818) | Scolytinae | 0 | 5 | 1 | x | - | no |
| *Scolytus scolytus* | (Fabricius, 1775) | Scolytinae | 0 | 3 | 1 | x | VU | no |
| *Taphrorychus bicolor* | (Herbst, 1793) | Scolytinae | 1848 | 0 | 1 | x | - | no |
| *Taphrorychus villifrons* | (Dufour, 1843) | Scolytinae | 0 | 5 | 1 | x | - | no |
| *Trypodendron domesticum* | (Linnaeus, 1758) | Scolytinae | 100 | 0 | 1 | m | - | no |
| *Trypodendron laeve* | Eggers, 1939 | Scolytinae | 1 | 0 | 1 | m | - | no |
| *Trypodendron lineatum* | (Olivier, 1795) | Scolytinae | 296 | 0 | 1 | m | - | no |
| *Trypodendron signatum* | (Fabricius, 1787) | Scolytinae | 0 | 7 | 1 | m | - | no |
| *Trypophloeus granulatus* | (Ratzeburg, 1837) | Scolytinae | 0 | 91 | 1 | x | - | no |
| *Xyleborinus alni* | Niijima, 1909 | Scolytinae | 3 | 22 | 1 | m | - | no |
| *Xyleborinus saxesenii* | (Ratzeburg, 1837) | Scolytinae | 1 | 82 | 1 | m | - | no |
| *Xyleborus cryptographus* | (Ratzeburg, 1837) | Scolytinae | 0 | 2 | 1 | m | - | no |
| *Xyleborus dispar* | (Fabricius, 1792) | Scolytinae | 16 | 29 | 1 | m | - | no |
| *Xyleborus dryographus* | (Ratzeburg, 1837) | Scolytinae | 0 | 16 | 1 | m | - | no |
| *Xyleborus monographus* | (Fabricius, 1792) | Scolytinae | 0 | 134 | 1 | m | - | no |
| *Xylechinus pilosus* | (Ratzeburg, 1837) | Scolytinae | 4 | 0 | 1 | x | - | no |
| *Anaspis flava* | (Linnaeus, 1758) | Scraptiidae | 0 | 28 | 1 | m | - | no |
| *Anaspis frontalis* | (Linnaeus, 1758) | Scraptiidae | 7 | 42 | 1 | m | - | no |
| *Anaspis melanostoma* | A. Costa, 1854 | Scraptiidae | 0 | 4 | 1 | m | VU | no |
| *Anaspis ruficollis* | (Fabricius, 1792) | Scraptiidae | 8 | 0 | 1 | m | - | no |
| *Anaspis rufilabris* | (Gyllenhal, 1827) | Scraptiidae | 151 | 2 | 1 | m | - | no |
| *Anaspis thoracica* | (Linnaeus, 1758) | Scraptiidae | 8 | 39 | 1 | m | - | no |
| *Scraptia fuscula* | P.W.J. Müller, 1821 | Scraptiidae | 0 | 5 | 1 | m | NT | no |
| *Phosphuga atrata* | (Linnaeus, 1758) | Silphidae | 0 | 2 | 3 | z | - | no |
| *Dendrophagus crenatus* | (Paykull, 1799) | Silvanidae | 1 | 0 | 1 | s | EN | yes |
| *Silvanus unidentatus* | (A.G. Olivier, 1790) | Silvanidae | 0 | 10 | 1 | s | - | no |
| *Uleiota planata* | (Linnaeus, 1761) | Silvanidae | 0 | 22 | 1 | s | - | no |
| *Aspidiphorus orbiculatus* | (Gyllenhal, 1808) | Sphindidae | 6 | 45 | 1 | m | - | no |
| *Sphindus dubius* | (Gyllenhal, 1808) | Sphindidae | 0 | 4 | 1 | m | - | no |
| *Allecula morio* | (Fabricius, 1787) | Tenebrionidae | 1 | 6 | 1 | s | - | no |
| *Allecula rhenana* | Bach, 1856 | Tenebrionidae | 0 | 1 | 1 | s | EN | yes |
| *Bolitophagus reticulatus* | (Linnaeus, 1767) | Tenebrionidae | 1 | 1 | 1 | m | - | no |
| *Corticeus bicolor* | (A.G. Olivier, 1790) | Tenebrionidae | 0 | 4 | 1 | s | - | no |
| *Corticeus unicolor* | (Piller & Mitterpacher, 1783) | Tenebrionidae | 1 | 5 | 1 | s | - | no |
| *Eledona agricola* | (Herbst, 1783) | Tenebrionidae | 0 | 1 | 1 | m | - | no |
| *Hymenophorus doublieri* | (Mulsant, 1851) | Tenebrionidae | 0 | 1 | 1 | m | CR | no |
| *Mycetochara axillaris* | (Paykull, 1799) | Tenebrionidae | 0 | 12 | 1 | m | - | no |
| *Mycetochara flavipes* | (Fabricius, 1792) | Tenebrionidae | 0 | 123 | 1 | m | VU | yes |
| *Mycetochara humeralis* | (Fabricius, 1787) | Tenebrionidae | 0 | 8 | 1 | m | - | no |
| *Mycetochara maura* | (Fabricius, 1792) | Tenebrionidae | 0 | 177 | 1 | m | - | no |
| *Mycetochara quadrimaculata* | (Latreille, 1804) | Tenebrionidae | 0 | 1 | 1 | m | CR | no |
| *Palorus depressus* | (Fabricius, 1790) | Tenebrionidae | 0 | 1 | 2 | s | NT | no |
| *Prionychus ater* | (Fabricius, 1775) | Tenebrionidae | 0 | 5 | 1 | s | - | no |
| *Prionychus melanarius* | (Germar, 1813) | Tenebrionidae | 0 | 1 | 1 | s | VU | yes |
| *Tribolium confusum* | Jacquelin du Val, 1861 | Tenebrionidae | 0 | 3 | 3 | s | - | no |
| *Uloma culinaris* | (Linnaeus, 1758) | Tenebrionidae | 0 | 2 | 1 | m | - | yes |
| *Hallomenus binotatus* | (Quensel, 1790) | Tetratomidae | 1 | 0 | 1 | m | - | no |
| *Tetratoma ancora* | Fabricius, 1790 | Tetratomidae | 12 | 0 | 1 | m | - | no |
| *Aulonothroscus brevicollis* | (Bonvouloir, 1859) | Throscidae | 24 | 47 | 3 | m | - | no |
| *Trixagus carinifrons* | (Bonvouloir, 1859) | Throscidae | 4 | 1 | 2 | m | - | no |
| *Trixagus dermestoides* | (Linnaeus, 1767) | Throscidae | 0 | 6 | 2 | m | - | no |
| *Trixagus elateroides* | (Heer, 1841) | Throscidae | 0 | 2 | 3 | m | - | no |
| *Trixagus gracilis* | Wollaston, 1854 | Throscidae | 0 | 3 | 3 | m | - | no |
| *Trixagus meybohmi* | Leseigneur, 2005 | Throscidae | 17 | 1 | 3 | m | - | no |
| *Trixagus obtusus* | (Curtis, 1827) | Throscidae | 0 | 9 | 3 | m | - | no |
| *Trox scaber* | (Linnaeus, 1767) | Trogidae | 0 | 1 | 2 | s | - | no |
| *Nemozoma elongatum* | (Linnaeus, 1761) | Trogositidae | 37 | 50 | 1 | z | - | no |
| *Thymalus limbatus* | (Fabricius, 1787) | Trogositidae | 1 | 0 | 1 | m | - | yes |
| *Aulonium trisulcum* | (Geoffroy, 1785) | Zopheridae | 0 | 1 | 1 | m | VU | yes |
| *Bitoma crenata* | (Fabricius, 1775) | Zopheridae | 1 | 4 | 2 | m | - | no |
| *Colydium elongatum* | (Fabricius, 1787) | Zopheridae | 0 | 5 | 1 | m | - | yes |
| *Colydium filiforme* | (Fabricius, 1792) | Zopheridae | 0 | 2 | 1 | m | VU | yes |
| *Rhopalocerus rondanii* | (A. Villa & J.B. Villa, 1833) | Zopheridae | 0 | 1 | 1 | m | EN | yes |
| *Synchita humeralis* | (Fabricius, 1792) | Zopheridae | 0 | 5 | 2 | m | - | no |
| *Synchita variegata* | Hellwig, 1792 | Zopheridae | 0 | 1 | 2 | m | EN | yes |
